# Automated bone marrow cell classification using ensemble learning: performance, generalization, and clinical interpretability

**DOI:** 10.3389/fmed.2026.1787948

**Published:** 2026-06-03

**Authors:** Shahid Mehmood, Muhammad Zubair, Sagheer Abbas, Rowida Mohammed Alharbi, Mai Alduailij, Muhammad Adnan Khan, Taher M. Ghazal

**Affiliations:** 1 Department of Computer Science, Bahria University, Lahore, Pakistan; 2 Department of Computer Science, Riphah International University, Islamabad, Pakistan; 3 Department of Computer Science, Prince Mohammad Bin Fahd University, Dhahran, Saudi Arabia; 4 King Abdulaziz City for Science and Technology, Institute of Earth and Space Sciences, Riyadh, Saudi Arabia; 5 Department of Computer Sciences, College of Computer and Information Sciences, Princess Nourah bint Abdulrahman University, Riyadh, Saudi Arabia; 6 Department of Software, Faculty of Artificial Intelligence and Software, Gachon University, Seongnam, Republic of Korea; 7 Department of Networks and Cybersecurity, Hourani Center for Applied Scientific Research, Al-Ahliyya Amman University, Amman, Jordan; 8 Faculty of Computing and IT, Sohar University, Sohar, Oman; 9 Center for Cyber Security, Faculty of Information Science and Technology, Universiti Kebangsaan Malaysia, Bangi, Selangor, Malaysia

**Keywords:** bone marrow cell classification, deep learning, ensemble learning, explainable AI, hematological diagnostics, medical image analysis, transfer learning

## Abstract

**Introduction:**

Bone marrow (BM) cell classification is essential for diagnosing a wide range of hematological disorders. However, automated classification remains challenging due to morphological overlap among cell types, class imbalance, and imaging artifacts. Although deep learning models, particularly convolutional neural networks (CNNs), have shown strong potential in medical image analysis, individual models may suffer from limited generalization and robustness.

**Methods:**

To address these limitations, we developed an ensemble-learning framework based on MobileNetV3 and ResNet18 to enhance feature extraction and classification performance while maintaining low computational cost. We evaluated four ensemble strategies: Soft Voting, Bagging, Boosting, and Stacking using a largescale dataset comprising more than 420,000 images across 21 bone marrow cell classes. External validation was performed using independent datasets to assess model generalizability under different imaging conditions. To improve interpretability, explainable AI methods, including Grad-CAM, Grad-CAM++, and LIME, were applied to visualize discriminative image regions. In addition, Decision Impact Ratio and Confidence Impact Ratio were used to quantify the reliability of the generated explanations.

**Results:**

Among the evaluated ensemble strategies, Boosting achieved the highest classification accuracy of 96%. External validation confirmed that the proposed model maintained robust performance across independent datasets and varying imaging conditions. The ensemble model outperformed individual models in both classification accuracy and interpretability stability. XAI analysis further demonstrated that the model focused on relevant morphological features, supporting the clinical plausibility of its predictions.

**Conclusion:**

The proposed MobileNetV3–ResNet18 ensemble framework provides accurate, computationally efficient, and interpretable bone marrow cell classification. By improving diagnostic performance and explanation reliability, this approach has strong potential to support AI-assisted hematological diagnostics, reduce diagnostic time, and minimize interobserver variability.

## 1 Introduction

BM is a very significant component of the body. It is a central location of cell production or hematopoiesis, in which red blood cells (oxygen carriers), white blood cells (infection fighters), and platelets (needed to form clots) are produced. The correct operation of the hematopoietic system is crucial to the fact that any person could be involved in the fight against the diseases and still in good health. Severe morbidity in terms of the health condition will occur due to any diseased condition of the BM whether it is leukemia, lymphoma or aplastic anemia, and therefore, the classification of BM cells is highly deemed critical in diagnosing and managing various diseases (1).

With many cells at various stages of development, all having only subtle morphological characters, identifying different types of BM cells, particularly rare, is challenging. The cells present in BM are diverse. Types of cells such as myeloblasts, promyelocytes, and monocytes can become very difficult at times to differentiate. Rarer types of cells, such as hairy cells or atypical eosinophils, are very scarce in the databases and considerably worsen identification and analysis. In addition, the existence of various artifacts during the microscopy process complicates the classification task, introducing intra-class variability and overlap in visual characteristics between different cell types (2).

Previously, medical image classification methods were not as competent and accurate as artificial intelligence (AI), especially deep learning (DL) methods. DL models can classify infections, fractures, neoplasia, and cardiac conditions using a variety of medical imaging modalities, such as MRI, CT scans, X-rays, ECG, and echocardiogram (3–6). In oncology, deep learning certainly finds its applications in the early detection of cancer (7–10), identification of circulating tumor cells (11, 12), and monitoring stem cell differentiations associated with regenerative medicine (13, 14). Hematology has been accelerated and made more accurate by AI systems. Identification using big data and advanced image analysis methods. These systems can provide an analysis of the complex morphology of BM (BM) cells in detecting in, essentially and crucially. BM-associated diseases include leukemia, lymphoma, etc. The automation assumes a lot of weight. The more traditional slow manual classification of off pathologists that is already afflicted with interobserver. Variability issues (15).

Features are learned and hierarchically extracted in convolutional neural networks (CNNs) with ease. Enable them to make proper distinctions between BM cell types. Studies have shown that CNNs have high classification accuracy, with some indicating that the performance of these models can be compared to that of a human expert. CNN has been used to classify acute lymphoblastic leukemia (ALL) cells in BM images with an accuracy greater than 97% (16). The transfer learning techniques have enhanced these models’ performance with fine-tuning parameters of pre-trained networks on smaller specialized medical datasets. This helps to overcome the most common data scarcity problems associated with medical imaging tasks (17).

Ensemble approaches refer to the methods in which different models are combined to improve certain areas of accuracy, robustness, and generalizing predictions regarding medical image classification. They aggregate and fuse outputs from the different models to minimize the risk of overfitted models and capture a larger variety of feature types that characterize medical images. Ensemble strategies include Bagging like Random Forests, boosting like XGBoost, and stacking. They help in better generalization by exploiting the merits of each one of these diverse classifiers (18, 19).

The bagging method minimizes the variance by constructing more than one training set through bootstrapping and models constructed in this way are useful in the high-variance models such as decision trees (20). It is highly effective in providing much more stable and confident estimations in small data or noisy situations in medical imaging. On the other hand, boosting builds powerful predictors by fusing multiple weak learners. These methods tend to be more than effective to enhance the accuracy of classification on the most difficult cases. Therefore, it is positive to strengthen such methods as AdaBoost and Gradient Boosting, which improve the classification of medical images, and it is more responsive and efficient when detecting less common diseases or abnormalities (21, 22). A stacking method involves training various base models and then developing a meta-model to integrate the predictions of the various models. By stacking medical image classifiers can be trained with exceptional strategies and architectures to enhance the interpretability and accuracy of the classifier across a wide range of datasets (23, 24).

Ensemble models are particularly appropriate in classification tasks of medical images that are needed to be highly precise and generalizing across varied datasets, for example in clinical settings. They have performed quite well compared to individual models in processes such as detection of cancer and diagnosis of medical scan diseases thus becoming attractive in terms of respective complex medical image analysis (25–27). The techniques have successfully been applied in various forms of application domains such as lung cancer detection in CT scan and histopathology images and have been consistently increasing accuracy and generalization performance on the tasks (28–30). Methods that use ensembles of multiple architectures in DL have reported improvements in the overall classification accuracy particularly of rare cell types or lower sample sizes (31).

The research gap, which is the focus of the study, is the lack of comparative study of the mechanisms of ensemble learning used in the classification of BM cells. Whereas CNNs and deep learning models have been successfully used in medical image analysis, the effectiveness of traditional methods of ensemble including voting, Bagging, boosting, and stacking in terms of their performance and generalization particularly in terms of rare types of BM cells, has not been strongly evaluated yet. Moreover, the influence of these ensemble methods on their generalization to other external datasets has not been widely researched in the case of hematological diseases, which is the objective of this study.

The following are the main contributions of this study:

1. *Integrated analysis of ensemble strategies*: A methodical comparison of four ensemble learning strategies Voting, Bagging, Boosting, and Stacking in bone marrow (BM) cell classification and their weaknesses and strengths.
2. *Extensive external validation*: The paper demonstrates the excellent performance in external validation of the model on two external datasets (BMCD-FGCD and A. Bodzas) that are independent of each other. The booster ensemble has the best score of 1.00 F1-score on the A. Bodzas dataset and this supports its superiority.
3. *Performance by class*: An in-depth performance analysis in classes, with confusion matrices, indicates that certain issues are observed with rare and morphologically similar BM cell types (e.g., Myeloblast vs. Promyelocyte).
4. *Feature representation analysis with UMAP*: Uniform Manifold Approximation and Projection (UMAP) is an algorithm that can be applied to analyze the visualization of high-dimensional feature embeddings to see how well ensemble models can distinguish BM cell types and where morphological overlaps can cause misclassifications.
5. *Explainability and interpretability*: This paper uses an extensive explainability model through the incorporation of Grad-CAM, Grad-CAM++, and LIME to improve the interpretability of the model. We have also used two new quantitative measures, Decision Impact Ratio and Confidence Impact Ratio, to make unbiased judgments regarding the reliability of such XAI methods. This visual and numerical confirmation of model decision-making offers the ability to discover diagnostically relevant regions and enhance a higher level of transparency in deep learning-based bone marrow cell classification.
6. *Computational efficiency optimization*: The research scales classification with computational efficiency such that boosting results in the highest accuracy (96%), and with a reasonable inference time (233.38 s) as well. The shortest testing time (22 s) is shown to be in stacking, yet it needs additional optimization of the meta-model.
7. *Medical AI and hematology significance*: The findings can be used to automate BM cell classification in hematological disorders that could aid in clinical and pathology operations. Future research might involve real-time implementation in digital hematology labs.
8. *Foundational benchmark for future research*: The study will be used as a benchmark study of ensemble learning in BM cell classification that will be used for developing future improvements in hybrid architectures, fined tuned ensemble selection and higher order approaches of feature fusion.

The rest of the paper is organized in the following way: Section 2 overviews the previous research and address associated literature on the topic of BM cell classification and ensemble learning methods. Section 3 indicates the methodology with the description of datasets, model architectures, and the applied ensemble strategies. In Section 4, the results of the experiment are provided, in which we compared the performance and generalization abilities of the ensemble methods. Section 5 covers the discussion of the findings and Section 6 brings the paper to a conclusion with some important implications and future research directions.

## 2 Related work

The historical classification of BM cell morphology has always been arduous because of the diversity of cell types, morphological similarity of the cell types, and high clinical diagnostic accuracy requirements. With the emergence of machine learning and deep learning in recent years, such techniques offer huge potential in automating and enriching this process. Ensemble learning has gained considerable momentum due to the fantastic capability of combining the strengths of many models to further increase performance and robustness. This section explains the related work in BM cell classification, ensemble learning methods, and possible future applications in medical image analysis.

### 2.1 Bone marrow cell classification using deep learning

There are studies that have been carried out to classify BM cells based on deep learning. A study by Jin et al. (28) was one of these studies that constructed an automated system of differentiating cell counts in BM smears with machine learning coupled with scanning equipment. The system could categorize with 600,000 images, and achieved 90.1% accuracy, and the highest results were achieved in the counts of granulocytes and erythrocytes. Their result with myeloid cells was, however, unsatisfactory because the stages of the cells were overlapping. Conversely, it performed poorly with unusual cell types such as eosinophils and basophils since there were few training results.

Wu et al. (29) created the BMSNet, a deep learning architecture, a combination of CNN and YOLO v3, used to identify and classify cells in BM smears. The model was trained on 122 smears of BM and in most categories had the same performance as hematologists. The model performed well in detecting blasts (AUC: 0.948 > 5% blasts) but was poor when blasts were above 20% because of variations in myelodysplastic syndrome cases. A deep learning system to automate BM cytology, identifying regions, detecting, and classifying cells from whole slide images was developed by Tayebi et al. (1). The system is characterized by the good region detection accuracy (0.97 accuracy, 0.99 ROC AUC) and solid results in cell classification (mean precision: 0.75, F1 score: 0.78). There are also limitations of overfitting, absence of external validation, and inability with rare or similar cell types. Lee et al. (30) created a deep learning algorithm based on InceptionV3 to categorize the BM smear cells in myelodysplastic syndromes (MDS). It exhibited high AUC (0.945–0.996) and accuracy (0.912–0.993) when it came to dysplastic cells but had lower sensitivity and precision than hematologists with inconsistent performance across cell types with dysplastic granulocytes excelling. Wang et al. (32) presented a hierarchical framework of the DL model to detect and classify 16 types of BM nucleated cells in whole slide images, including infrequent ones such as megakaryocytes and mitotic cells. The framework had high recall rates (>0.9 of 11/17 cell types) but had a restrictive small dataset of 12,426 images of 27 patients. In a similar fashion, Ananthakrishnan et al. (33) put forward a Siamese neural network to automatically classify BM cells. Their model with a dataset size of 170,000 expert-annotated cell images reached a training accuracy of 91% and a validation accuracy of 84% with useful results to issues of class imbalances.

Recent prospective clinical studies have begun to bridge the gap between technical benchmark performance and real-world clinical utility. Matek et al. (34) demonstrated human-level accuracy for blast cell recognition in AML using a CNN trained on the MLL Munich dataset — the same dataset used in the present study validated prospectively against five hematopathologists, establishing a human performance baseline directly applicable to the present work. The boosting ensemble evaluated in the present study achieves a macro F1-score of 0.96 across all 21 cell classes, which compares favorably to the reported inter-expert agreement of 85–91% for morphologically challenging cell types (34, 35).

Goldgof et al. (36) conducted the most comprehensive prospective clinical validation to date of a BM cell AI system (DeepHeme), evaluating performance against 40 hematopathologists across five academic medical centers on 23 morphological classes. DeepHeme achieved median performance statistically non-inferior to the median expert across most cell classes, establishing the target benchmark for clinical readiness of systems like the one proposed hereThe agreement thresholds and clinical outcome metrics reported in these prospective studies now define the validation standards that future iterations of the present system must meet before regulatory submission.

A critical challenge for generalizing BM cell classification models beyond single-institution training is the privacy-preserving aggregation of multi-site image data. Federated Learning (FL), in which models are trained locally at each institution and only weight updates are shared with a central aggregator, offers a principled solution to this challenge. Sarma et al. (37) demonstrated that federated training across six medical centers improves classification generalization compared to single-center training, approaching the performance of centralized pooled data without sharing any patient images. Sarma et al. (38) extended FL to whole-slide digital pathology, showing feasibility for large-scale histopathology model training across institutions with heterogeneous imaging protocols. Linardos et al. (39) provided a simulation framework for evaluating FL performance degradation under realistic data heterogeneity conditions. Together, these studies establish FL as a technically mature approach for multi-institutional BM classification training, an important future direction for the system proposed in the present study, where the primary limitation is the reliance on a single-institution training dataset.

### 2.2 Hybrid models for bone marrow cell classification: advances in CNN and transformer integration

Recent years have witnessed a rapid shift from purely convolutional architectures toward transformer-based and hybrid models for hematological image analysis. Vision Transformers (ViT) and their variants leverage self-attention mechanisms to capture long-range spatial dependencies within cell images, providing a complementary inductive bias to the local feature extraction of CNNs. Indicatively, Glüge et al. (40) assessed 4 CNN models using 171,374 images to improve BM cell classification. The Regnet_y_32gf model performed better than ResNeXt-50 with precision, recall and F1 score of 0.787, 0.755 and 0.762 when trained out-of-domain. Class activation maps effectively identify diagnostic features like Auer rods. Other models such as VGG-19 BN and ResNet-152 were more efficient compared to ResNeXt-50 whereas ViT_l_32 underperformed as it requires huge datasets. Acevedo et al. (41) compared CNN architectures (ResNet, EfficientNet) with Swin Transformer on peripheral blood cell classification, finding that Swin Transformer achieved superior F1-scores for morphologically similar cell pairs including band versus segmented neutrophil and eosinophil versus basophil precisely the cell type pairs that present the greatest challenge in the present study. Zhao et al. (42) proposed a dual-branch CNN–Transformer architecture targeting the myeloblast–promyelocyte–metamyelocyte disambiguation problem, demonstrating improved precision at maturation-stage boundaries. Transformer models achieve superior performance for fine-grained morphological discrimination but require substantially larger annotated datasets than CNNs to realize their advantage, a constraint that remains significant in the hematological imaging domain where expert-annotated datasets remain limited. These findings position the CNN ensemble evaluated in the present study as the robust, data-efficient solution for current dataset scales, while motivating transformer integration as a priority direction for future large-scale studies.

Tripathi et al. (43) introduced a solution to the classification of BM cells with the use of data augmentation to resolve the problem of unbalanced and small datasets. Because of its attention mechanism, CoAtNet model was more precise and more recall-oriented than EfficientNetV2, and more moderate in accuracy (0.82), as it failed with some classes of BM cells. Peng et al. (44) developed the Dual Attention Gates DenseNet (DAGDNet), which had added the idea of dual attention to the DenseNet architecture. DAGDNet, which was trained on a dataset that was mostly comprised of leukemia samples, performed better than conventional models such as DenseNet and ResNeXt, with a mean precision of 88.1% on the Munich Leukemia Laboratory dataset. Chen et al. (45) have come up with SCKansformer, a fine-grained BM blood cell classification model. The model combines the Kansformer Encoder, SCConv Encoder, and Global–Local Attention Encoder to increase the accuracy and efficiency of classification, and it is superior in both private and public datasets.

### 2.3 Ensemble learning in medical image analysis

The concept of ensemble learning has been well-known to enhance the classification accuracy through the combination of the predictions of numerous models. Voting, Bagging, boosting, stacking among other techniques have been used in different medical imaging tasks. To illustrate, Kini et al. (46) suggested an ensemble deep learning and IoT model which utilizes medical IoT devices to gather CT and automates COVID-19 diagnosis with the high accuracy (98.98%) and outperforming 13 competitive models in precision, recall, F1-score, and AUC. On the same note, Maftouni et al. (47) present an efficient COVID-19 classifier on noisy labels of chest CT scans based on an ensemble model consisting of Residual Attention and DenseNet architectures.

The EDLCDS-BCDC method proposed by Ragab et al. (48) relied on the ultrasound images of the breast to diagnose cancer through wiener filtering, contrast enhancement, CKHA-based segmentation, and an ensemble of VGG-16, VGG-19, and SqueezeNet to extract features. Likewise, ensemble approaches have been used to analyze histopathological images, where complex patterns and variability in staining make individual models prone to overfitting. Zheng et al. (49) suggest a deep ensemble model on the basis of image-level labels, data augmentation, and four pre-trained networks (VGG16, Xception, ResNet50, DenseNet201) to classify benign and malignant breast histopathological images in the BreaKHis dataset on a binary basis. The multi-model and multi-slice architecture with 2D CNNs introduced by Kang et al. (50) to classify Alzheimer disease is an ensemble learning architecture, which consists of VGG16, ResNet50, and GAN discriminator.

### 2.4 Ensemble learning for bone marrow cell classification

Although ensemble learning was considered in more general cases of medical imaging, its implementation to BM cell classification has been relatively small. Another interesting study is the one by Walid et al. (51), where a new residual CNN (including or without LSTM) was proposed as a classifier of BM cells into 21 categories and significantly exceeds 11 variants of deep-transfer learning. The combination of the top 3 models (max voting ensemble) has the highest kappa score (86.29%), then Residual CNN-LSTM (86.11%), and EfficientNetB3 (85.10%).

### 2.5 Challenges and gaps

Regardless of the progress in ensemble learning and the classification of BM cells, there are still a number of difficulties. The identification and formulation of the best ensemble strategies have not been well investigated, especially in diverse cell types and with external validation data. Also, no research has been done on the comparison of effects of various ensemble approaches, e.g., voting, Bagging, boosting and stacking on the classification performance and generalization. Moreover, XAI is not actively incorporated into ensemble models, and the ambiguities about understanding trade-offs between accuracy and interpretability have not been assessed in a systematic manner.

## 3 Methodology

The study will assess and compare ensemble learning approaches for classifying BM cell morphology, such as Bagging, boosting, and stacking. The aim is to identify the best of these strategies for hematologic diagnostics in terms of accuracy, generalizability, and employability in imbalanced and complex dataset conditions.

### 3.1 Dataset description

The study is based on a dataset of high-resolution microscopic images of BM cells belonging to 21 different cell types (52). Experienced hematologists carefully labeled each image, providing the ground-truth annotations necessary for supervised learning. The final dataset was preprocessed and augmented to improve the model’s generalization ability across different cell morphologies and imaging conditions. 945 BM cytological samples were taken from patients with hematological disorders between 2011 and 2013 at MLL Munich Leukemia Laboratory. According to the Declaration of Helsinki, clinical data was used after written informed consent had been obtained from all patients. A single-cell image cannot be used to track a specific patient. An internal institutional review board at MLL Munich Leukemia Laboratory approved the study.

With a median of 69.3 years and a mean of 65.6 years, the patients included in the study ranged in age from 18.1 to 92.2 years. Among the cohort, 59.8% of the patients were males, and 40.1% were females, with 0.1% being of unknown gender. The original dataset consisted of 171,374 samples, which underwent preprocessing and augmentation to address class imbalance and augment feature diversity.

### 3.2 Dataset augmentation and balancing strategy

The problem of imbalance among the classes, which is in the first place of machine learning and is especially relevant during the classification tasks, results in the training of skewed models that perform poorly in the least represented classes but well with dominant ones. This issue is critical in the field of medical diagnostics, and in this case, the classification of the least represented classes becomes critical. Imbalanced data can compromise evaluation metrics like accuracy, such that usually a model may be described as performing well overall while still performing poorly on minority classes. The imbalance may also result in underfitting due to the inadequacy of minority class data or overfitting on the available few examples. The deep learning models in general, and particularly the CNNs, are very much prone to face these issues, since if there is even more imbalance, the performance on the minority class deteriorates even further (53, 54).

The original MLL dataset (171,374 images across 21 cell types) was first divided into training and test sets using an 80/20 stratified image-level split, applied to the original, pre-augmentation images. The test set was held out in its original, unaugmented form throughout all experiments and was used exclusively for final evaluation. No test images were used for any model training, hyperparameter selection, or early stopping decisions. The publicly released version of the MLL dataset [(34), TCIA] does not include patient-level identifiers, as patient metadata was excluded from the release to protect privacy under the Declaration of Helsinki consent framework. Consequently, patient-level splitting was not possible with the available data. Image-level stratified splitting was applied. A combination of data augmentation, oversampling, and under-sampling techniques were used to balance the training set, to 20,000 images per class. This has been widely referenced in literature with respect to improving a model’s performance and generalization, especially in a multiclass scenario with extreme imbalances (35, 55–57).

### 3.3 Model architectures and ensemble strategies

In this research, four strategies of ensemble learning were assessed, namely, soft voting, Bagging, boosting, and stacking. The complementary architectural advantages of the two basic models, ResNet18, and MobileNetV3, determined its selection as the two models complement the ensemble approaches to accuracy, performance, and the capacity to extract features. All the models are explained below with respect to the theoretical justification:

#### 3.3.1 ResNet18

The simple form of ResNet architecture, ResNet18 (58), has powerful deep learning properties since it does not fall into the traps of the vanishing gradient problem by making use of a residual connection. The properties are essential in the medical image classification, in which slight morphological variations in the types of cells demand expressive feature representations. According to the theory of ResNet, due to the skip connections, the gradients are allowed to pass through the deeper layers without losing their learning capacity, increasing convergence. The justifications for choosing ResNet18 include its ability to capture high-level morphological features of BM cells, efficient training due to its relatively small number of parameters compared to deeper ResNet variants, and proven success in medical image analysis, including pathology and histopathology classification tasks.

#### 3.3.2 MobileNetV3

MobileNetV3 (59) is a CNN that is lightweight and optimized for edge and mobile computing. Depth separable convolution is used to lower the computational cost while retaining high accuracy. Furthermore, Squeeze-and-Excitation (SE) blocks improve performance by further focusing on important spatial features. MobileNetV3 was selected because it significantly reduces inference time, making it suitable for large-scale classification tasks. Depth wise separable convolutions improve efficiency while preserving accuracy. It complements ResNet18 by making the feature extraction process much more efficient, which, in essence, means that the whole ensemble will be robust and computably feasible.

#### 3.3.3 Soft voting ensemble

The soft voting ensemble method averages the probability predictions given by several base models to reach the final decision about the class assignment. Let *M_1_, M_2_,…, M_n_* represent the *n* base models in the ensemble, where each probability distribution *P_i,c_* is defined over the classes *c* for the given input sample *x*. We will average the likelihoods for class *c* and compute *P_c_* by the following formula as shown in Equation 1:


$$P_{c}=\frac{1}{n\;\;}\;\sum_{i=1}^{n}P_{i,c}$$ (1)

Here, $\;P_{i,c}$ refers to the probability assigned to class *c* by the *i-th* model, and n is the number of models in the entire ensemble. The label $\hat{Y}$ is finally inferred by taking that class with the highest combining probability as shown in Equation 2:


$$\hat{Y}=\text{argmax}\max_{c}P_{c}$$ (2)

**Figure 1:**
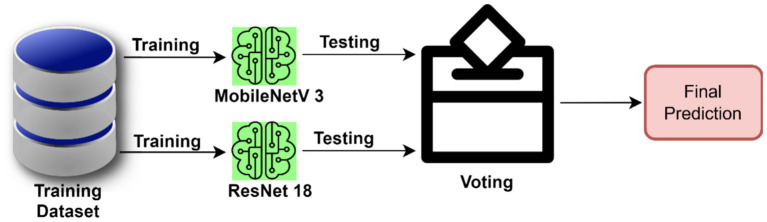
Implementation of soft voting in the ensemble model, combining probabilistic outputs from ResNet18 and MobileNetV3 for final bone marrow cell classification.

Figure 1 illustrates the implementation of soft voting in the ensemble model used for BM cell classification. Soft voting combines the probabilistic outputs from multiple models, in this case, ResNet18 and MobileNetV3, to make a final prediction. Each model contributes its confidence scores for different cell classes, and the final prediction is determined by averaging these probabilities. This method combines strengths of both architectures and may result in a more reliable and accurate classification. It enhances the functioning and reliability of the model, by reducing the individual model biases and flaws.

#### 3.3.4 Bagging ensemble

In this work, Bagging or Bootstrap Aggregating was employed to make the performance of the BM cell morphology classification better by reducing the variance of the model and enhancing the generalization. In this aspect, the bagging ensemble has been implemented using two base models, MobileNetV3 and ResNet18 to enhance their robust and accurate predictions.

**Figure 2:**
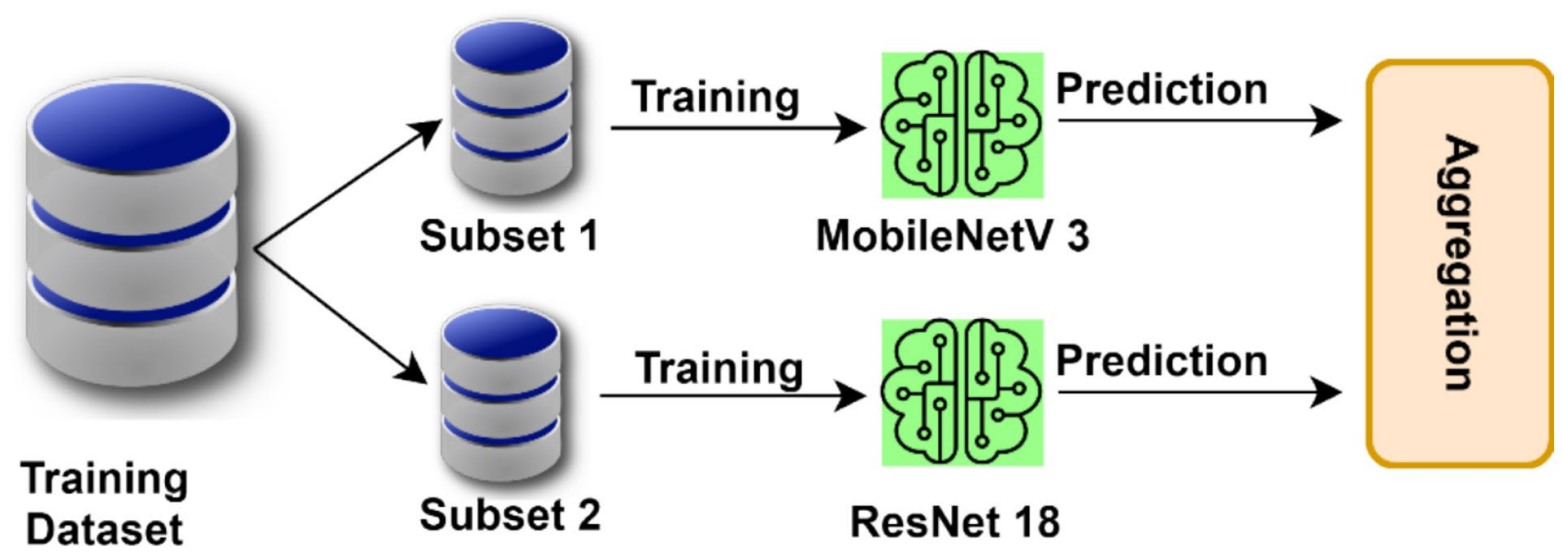
Examples of the bagging process in the ensemble model, featuring the various models that are trained separately on various subsets of the data. All the models come up with their own predictions, and the outcomes are combined to come up with a more stable prediction.

Bootstrap sampling was applied to generate various subsets of the training data on each base model that ensured that all the subsets were sampled with replacement thereby ensuring variability in the input to the base models as represented in Figure 2. The two basic models were trained separately using their respective bootstrap samples. The two models were fine-tuned with the help of transfer learning to adjust the pre-trained weights of the models to the BM cell dataset. The ADAM was used to optimize the models, early stopping and schedule learning rates were used to encourage the high performance of the models without causing the overfitting problem.

After training, each base model Mi (M1 in the case of MobileNetV3 and M2 in the case of ResNet18) made predictions of classes $\hat{y}$ of an input sample *x*. The majority voting was done to determine the final prediction $\hat{y}$ of the ensemble which was based on summing the class predictions of the two models as shown in Equation 3:


$$\hat{y}=\arg\max_{c}\;\sum_{i=1}^{2}\;1({\hat{y}}_{i}=c)$$ (3)

In this case, $1({\hat{y}}_{i}=c)$ is a function that is equal to 1 when the prediction of model *Mi* is class *c* and *0* otherwise. Majority voting will make the ensemble prediction represent the agreement of the base models.

The variance reduction effect of ensemble learning is often explained under simplifying assumptions. For an ensemble of n models with identical variance and uncorrelated predictions, the variance of the averaged prediction can be expressed as in Equation 4:


$$\mathrm{Var}(\hat{y})=\frac{1}{n}\;\mathrm{Var}({\hat{y}}_{i})$$ (4)

This idealized formulation suggests that combining multiple models reduces prediction variance proportionally to the number of ensemble members. For example, in the case of two independent models, the variance is reduced by half. However, this assumption does not strictly hold in practical scenarios. In real-world settings, base models are often trained on overlapping data distributions (e.g., bootstrap samples from the same dataset) and therefore exhibit positive correlation in their predictions. Taking this into account, the variance of the ensemble can be more accurately expressed as in Equation 5:


$$\mathrm{Var}(\hat{y})=\frac{1+(n-1)\rho }{n}\;\mathrm{Var}({\hat{y}}_{i})$$ (5)

Where ρ represents the average pairwise correlation between base models. This formulation highlights that the effectiveness of ensemble learning depends not only on the number of models but also on their diversity. When ρ=0 , the ensemble achieves maximal variance reduction. As ρ increases, the benefit diminishes, and for highly correlated models, the variance reduction becomes limited. In this study, although the base models are trained on related data, diversity is introduced through differences in network architectures (MobileNetV3, ResNet18), optimization dynamics, and stochastic regularization techniques such as dropout. These factors help reduce correlation between models and contribute to improved generalization performance.

#### 3.3.5 Boosting ensemble

**Figure 3:**
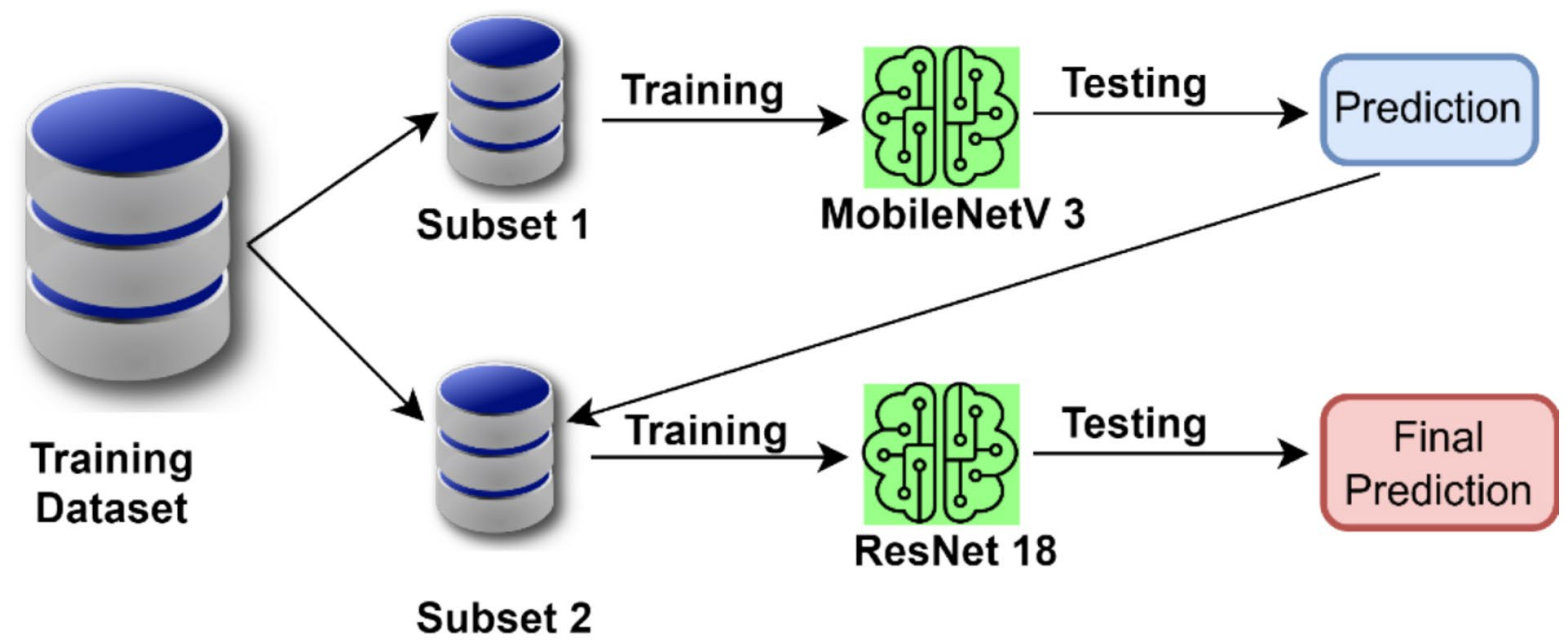
Example of the process of boosting in the ensemble model that demonstrates the sequential improvement of predictions to increase the ultimate accuracy of classification in identifying bone marrow cells.

In the boosting model as shown in Figure 3, once the first model had been trained the errors (misclassifications) that the first model made were examined and a weight adjustment mechanism was used to focus the attention of the second model training on the errors of the first model. The boosting ensemble implemented in this study is an AdaBoost-inspired sequential ensemble framework adapted for deep convolutional neural networks. Classical AdaBoost was designed for weak learners that accept per-sample weights directly during training. Since deep neural networks trained with standard gradient-based optimizers do not natively support per-sample weight injection, the boosting adaptation in this work incorporates sample weights through a weighted cross-entropy loss function applied to the second sequential model, consistent with established practice for deep ensemble learning (31).

The sequential training procedure was executed in four stages: Stage 1. MobileNetV3 (Model 1) was trained on the full balanced dataset with uniform sample weights using cross-entropy loss and Adam optimization (see Section 3.4). Stage 2. Inference was run on the training set using the trained Model 1. Misclassified samples were identified using the indicator function 1 (yᵢ ≠ ŷᵢ). Stage 3. Sample weights were updated and normalized. For each training sample i, the weight was updated as shown in Equation 6:


$$w_{i}^{t+1}=w_{i}^{t}\cdot \exp(\alpha_{t}\cdot 1(y_{i}\neq {\hat{y}}_{i}))$$ (6)

Where w_i^t is the weight of the i-th sample at iteration t, α_t is the model weight for Model 1 (derived below), yᵢ is the true label, ŷᵢ is the predicted label, and 1(·) is the indicator function. After updating, weights were normalized to sum to 1, as shown in Equation 7:


$$w_{i}^{t+1}\leftarrow \frac{w_{i}^{t+1}}{\;\;\sum_{j}w_{i}^{t+1}}$$ (7)

The model weight α_t for Model 1 was computed from its weighted training error ε_t as follows as shown in Equation 8:


$$\begin{array}{c} \varepsilon_{t}=\sum_{i=1}^{N}\;w_{i}^{t}\cdot 1(y_{i}\neq {\hat{y}}_{i})\;\\ \alpha_{t}=\frac{1}{2}\ln(\frac{1-\varepsilon_{t}}{\varepsilon_{t}}) \end{array}$$ (8)

A model with lower training error (smaller ε_t) receives a higher weight α_t, reflecting greater contribution to the final prediction. Stage 4. ResNet18 (Model 2) was trained using the normalized sample weights from Stage 3, incorporated as per-sample scaling factors in the cross-entropy loss as shown in Equation 9:


$${ℒ}_{\text{weighted}}=-\sum_{i=1}^{N}\;w_{i}\cdot \log P_{2}(y_{i}|x_{i})$$ (9)

This ensures that Model 2 focuses proportionally more on samples that were misclassified by Model 1. The core operational principle of boosting. This approach to deep network boosting is consistent with Ren et al. (60) and prior deep CNN ensemble literature (31). After both models were trained, their predictions were combined using a normalized weighted averaging approach. The ensemble output for an input sample x as shown in Equation 10:


$$P_{\text{ensemble}}(c|x)=\frac{\sum_{i=1}^{n}\;\alpha_{i}\cdot P_{i}(c|x)}{\sum_{i=1}^{n}\;\alpha_{i}}$$ (10)

Where Pᵢ(c|x) is the probability of class c predicted by the i-th model, αᵢ is the model weight determined by its weighted training error, n is the total number of models, and the denominator Σᵢ αᵢ normalizes the ensemble output to a valid probability distribution. The final predicted class is:

The final prediction was the class with the highest ensemble probability as shown in Equation 11:


$${\hat{y}}_{\text{ensemble}}=\arg\max_{c}\;P_{\text{ensemble}}(c|x)$$ (11)

It is important to note that the present implementation employs two deep CNN learners rather than the many weak learners used in classical AdaBoost. This design decision was motivated by the substantial computational cost of training large-scale deep networks on over 420,000 images, and by the architectural diversity between MobileNetV3 and ResNet18 (depth-separable convolutions vs. residual skip connections), which ensures the inter-model diversity required for effective boosting without requiring additional training rounds. The empirical results confirm that meaningful sequential error correction occurred: the boosting ensemble achieved 96% accuracy, outperforming all other ensemble strategies evaluated (voting: 94%, bagging: 93%, stacking: 94%). Future work will investigate multi-round deep boosting with additional architectures to further exploit the iterative error-correction mechanism.

#### 3.3.6 Stacking ensemble

**Figure 4:**
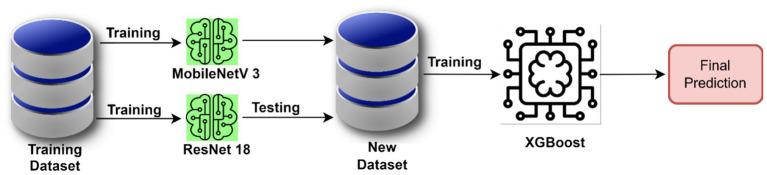
Visualization of the stacking method in the ensemble model, where predictions from base models (ResNet18 and MobileNetV3) are combined using a meta-classifier to produce the final bone marrow cell classification.

Stacking is an advanced ensemble learning technique that combines predictions from multiple base models using a meta-model, which learns the optimal way to aggregate their outputs. In this research, MobileNetV3 and ResNet18 were used as base models for their complementary strengths, with XGBoost as the meta-model to improve the classification of BM cells into 21 distinct categories, as illustrated in Figure 4. Each model’s final classification layer was replaced with a fully connected layer tailored to the 21-class BM dataset. Both models were trained individually on the same dataset using cross-entropy loss and Adam optimization.

For an input sample *x*, each base model $M_{i}$ ( i=1,2 ) outputs a prediction vector as shown in Equation 12:


$$P_{i}=[P_{i.1},P_{i.2},\ldots ,P_{i.C}]$$ (12)

Where $P_{i.C}$ is the probability assigned to class C by the i *-th* model, and *C = 21* represents the total number of classes. The outputs $P_{1}$ and $P_{2}$ from the two base models are concatenated to form a new feature vector *Z* as shown in Equation 13:


Z=[P1,P2] (13)

In this case, *Z* is a 2 × 21-dimensional vector (flattened to 42 features). The concatenated feature vector *Z* serves as input to the XGBoost meta-model, which learns to combine the strengths of the base models by minimizing a classification loss function (e.g., log loss). The meta-model generates probabilities $P_{\text{meta}}\;(c|x)$ for each class c: as shown in Equation 14:


$$P_{\text{meta}}\;(c|x)=\text{XGBoost}(Z)$$ (14)

The final predicted class label $\hat{Y}$ is obtained by selecting the class with the highest probability as shown in Equation 15:


$$\hat{Y}=\arg\max_{c}P_{\text{meta}}\;(c|x)$$ (15)

The XGBoost classifier was configured with the following hyperparameters: number of trees (n_estimators) = 100, maximum depth (max_depth) = 3, learning rate (*η*) = 0.1, subsample ratio = 0.8, and column subsampling ratio (colsample_bytree) = 0.8. The objective function was set to *multi:softprob*, and the evaluation metric was *mlogloss*. A fixed random seed (42) was used to ensure reproducibility.

### 3.4 Experimental setup

All experiments were conducted on a MacBook Pro with an Apple M1 Pro chip featuring a 10-core CPU, 16-core GPU, and 32 GB of RAM. This setup offered enough computing power to train deep learning models and work with a big set of BM cell images. The PyTorch framework was used to train the models with the help of the Metal Performance Shaders (MPS) backend optimized to run on the M1 chip. Acceleration of processing was done using the GPU to ensure that the MobileNetV3 and ResNet18 models were trained at a very high rate during the ensemble framework.

### 3.5 Training configuration

All deep learning models were trained using the Adam optimizer with an initial learning rate of 0.001 and a cosine annealing learning rate schedule. A batch size of 128 was used for all experiments. Training was conducted for a maximum of 10 epochs, with early stopping applied using a patience of 3 epochs based on validation loss. In practice, most models converged within 6–8 epochs, preventing overfitting while maintaining computational efficiency.

### 3.6 Evaluation metrics

The model’s effectiveness in classifying BM cell types has been accessed using important performance metrics- precision, recall, F1-score, and accuracy. These have all been used in image classification, especially in medical imaging, as they give insight into the correctness of prediction by the model concerning some errors. Below is a description of each metric with its mathematical formulation:

Precision measures how many of the instances predicted as positive are correct, as shown in Equation 16. It gives us an idea about the model’s ability to minimize false positives.


$$\text{Precision}=\frac{\mathrm{TP}}{\mathrm{TP}+\mathrm{FP}}$$ (16)

Recall, also known as sensitivity or true positive rate, measures how well the model identifies actual positive cases, as shown in Equation 17. High recall is more important in medical applications because false negatives or missing a positive case may have serious consequences.


$$\text{Recall}=\frac{\mathrm{TP}}{\mathrm{TP}+\mathrm{FN}}$$ (17)

F1-score is defined as the harmonic mean of precision and recall, thus providing a proper measure only in cases where an imbalance exists between precision and recall, as shown in Equation 18. In cases where false positives and false negatives must be greatly reduced, the F1-score is preferred.


$$F1=\frac{\mathrm{TP}}{\mathrm{TP}+\frac{1}{2\;}\;(\mathrm{FP}+\mathrm{FN}}$$ (18)

Accuracy assesses how good a model is, evaluating the ratio of correct predictions (true positives + true negatives) against all predictions made, as shown in Equation 19. While an important metric, it can be rather misleading when working with an imbalanced dataset; hence, precision, recall, and F1-score should be used for a more extensive evaluation.


$$\text{Accuracy}=\frac{\mathrm{TP}+\mathrm{TN}}{\mathrm{TN}+\mathrm{TP}+\mathrm{FN}+\mathrm{FP}}$$ (19)

## 4 Results and discussion

This section focuses on results and discusses in depth the performance of various ensemble learning strategies applied to BM cell morphology classification. Important results with class-wise metrics and generalization capabilities of each method are described to highlight strengths and limitations. The explainable AIs and their role in the process of decisions made by the models are also discussed. This part should provide the clue to the relevance and effectiveness of ensemble strategies in hematological diagnostics.

### 4.1 Results

This section provides the findings of the experiments applicable to the MLL dataset with the application of the various ensemble techniques, such as Voting, Bagging, boosting, and stacking.

#### 4.1.1 Standalone base model performance

Prior to presenting the ensemble results, we report the standalone classification performance of each individual base model (MobileNetV3 and ResNet18) on the MLL test set, which serve as the primary baselines against which the incremental benefit of ensemble combination is measured. Both models were evaluated under identical conditions: same training data, hyperparameters, and test set as the ensemble configurations. Results are summarized in Table 1 alongside all four ensemble methods for direct comparison. MobileNetV3, evaluated as a standalone classifier, achieved an accuracy of 88.41% and a macro F1-score of 0.8849. ResNet18 performed considerably better individually, achieving 91.47% accuracy and a macro F1-score of 0.9152, consistent with its deeper residual architecture and superior capacity for fine-grained texture feature extraction. The performance gap between the two base models (*Δ* accuracy = +3.06%, McNemar’s *p* < 0.0001, Cohen’s h = 0.113) confirms that ResNet18 is the stronger backbone — an important baseline context for interpreting the ensemble results.

**Table 1:** Complete performance comparison of individual base models (MobileNetV3, ResNet18) and all four ensemble strategies on the MLL test set.

Model/Method	Accuracy	Precision	Recall	F1-Score	Macro ROC-AUC
Individual base models					
MobileNetV3 (individual)	0.8841	0.8863	0.8841	0.8849	0.9912
ResNet18 (individual)	0.9147	0.9163	0.9147	0.9152	0.9941
Ensemble methods					
Soft Voting	0.9400	0.9401	0.9400	0.9400	0.9961
Bagging	0.9300	0.9314	0.9300	0.9304	0.9948
Boosting (Best)	**0.9600**	**0.9601**	**0.9600**	**0.9600**	**0.9978**
Stacking	0.9400	0.9402	0.9400	0.9400	0.9958

Bold values indicate the best-performing result for each evaluation metric across all individual and ensemble models.

Critically, all four ensemble strategies outperform ResNet18 individually, demonstrating that ensemble combination provides genuine incremental value beyond the stronger backbone alone. The boosting ensemble achieves the largest absolute gain over ResNet18 (+4.53% accuracy, +0.0448 macro F1, McNemar’s *p* < 0.0001, Cohen’s h = 0.171), demonstrating that the sequential error-correction mechanism which explicitly re-weights training samples misclassified by MobileNetV3 before training ResNet18 extracts substantial additional discriminative capacity beyond what either architecture achieves independently.

#### 4.1.2 Soft voting results

The ensemble model of MobileNetV3 and ResNet18 has good outcomes with respect to the overall BM cell classification with a total score of 94 as shown in Table 2. The model also has almost 100 percent accuracy, recall and F1 scores with most cell types such as ABE, BAS, FGC, HAC, KSC, LYI and OTH which have a score of 1 on all the performance measures. The model is able to forecast those categories with absolute or 100 percent accuracy and no classification errors. The findings indicate that the light architecture of MobileNetV3 can be merged with the strong extracting feature of ResNet18 with ease to proficiently categorize BM cells.

**Table 2:** The performances of the soft voting ensemble model (MobileNetV3 + ResNet18) on BM cell classification.

Cell type	Precision	Recall	F1-score	Support
ABE	1.00	1.00	1.00	4015
ART	0.92	0.89	0.90	4050
BAS	0.99	1.00	1.00	3983
BLA	0.87	0.85	0.86	4010
EBO	0.98	0.98	0.98	3887
EOS	0.99	0.98	0.99	3983
FGC	1.00	1.00	1.00	3999
HAC	0.99	1.00	1.00	3963
KSC	1.00	1.00	1.00	3997
LYI	1.00	1.00	1.00	3970
LYT	0.98	0.98	0.98	4021
MMZ	0.82	0.84	0.83	4143
MON	0.89	0.90	0.90	3961
MYB	0.79	0.81	0.80	3966
NGB	0.89	0.86	0.88	4049
NGS	0.99	0.99	0.99	4026
NIF	0.89	0.92	0.91	3896
OTH	1.00	1.00	1.00	4009
PEB	0.95	0.96	0.96	4050
PLM	0.96	0.96	0.96	3984
PMO	0.81	0.80	0.81	4038
Accuracy			0.94	84000
Macro avg.	0.94	0.94	0.94	84000
Weighted avg.	0.94	0.94	0.94	84000

The ensemble model performed better with most classes than with single classifiers, with the other metrics being evenly spread over their scores, as well, with 0.94 macro and weighted average precision, recall and f1 -scores. The types of cells ART, MON and NGB were almost even with f1-scores of 0.88 to 0.90. These results indicate that the ensemble model is very well applicable in different cell types, which makes it useful in cell classification, particularly in the state-of-the-art performance in several cell types.

**Figure 5:**
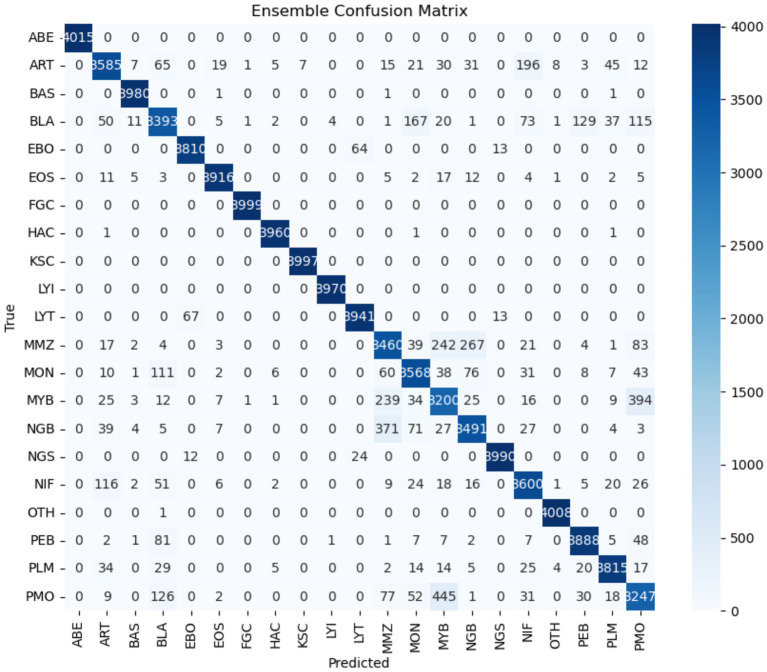
Soft voting ensemble model confusion matrix of BM cell classification.

The ensemble model combined with MobileNetV3 and ResNet18 in Figure 5 has a confusion matrix that indicates that the model is doing very well in the classification of the different BM cell types. The correct rates of the actual labels are concentrated mostly along the diagonal meaning that most of the classes have a high correct classification rate. Specifically, ABE, BAS, FGC, and KSC types of cells show almost or ideal classification outcomes of these classes as indicated by the strong diagonal line. The model works quite well with HAC, LYI, and EOS with a very limited number of cells being misclassified. It is implied that important BM cell types, which are significant to clinical diagnosis applications, can be identified with great precision by the ensemble model where specific cells are required.

The confusion matrix confirms strong overall performance, with inter-class confusion limited mainly to morphologically similar types such as BLA and MMZ. The soft voting ensemble effectively leverages the complementary strengths of MobileNetV3’s efficiency and ResNet18’s deep feature extraction, yielding balanced classification performance across all 21 cell classes.

Though our ensemble model had a high overall level of performance, there were some exceptional cell types with lower F1-scores including promyelocytes (PMO). The reason could be the morphological similarity with other classes, which might require higher-level features extraction or a greater number of samples of the dataset on these rarer classes. In addition, the ensemble method added to the accuracy of detection, but alleviated the complexity of the computation, which can be refined in the future by taking more efficient architecture into consideration.

#### 4.1.3 Bagging results

Almost all types of cells work well with the bagging ensemble method which lowers the variance and handles the class imbalance. It provided high accuracy, precision, recall and F1 values, thus it established reliability in the BM cell classification. Table 3, further addressed below, summarizes performance metrics per cell type.

**Table 3:** Class-wise performance of the ensemble model on BM cell classification using bagging technique.

Cell type	Precision	Recall	f1-score	Support
ABE	1.00	1.00	1.00	4029
ART	0.89	0.90	0.89	4002
BAS	0.99	1.00	0.99	4053
BLA	0.86	0.85	0.85	4045
EBO	0.98	0.98	0.98	4058
EOS	0.99	0.98	0.98	4104
FGC	1.00	1.00	1.00	3941
HAC	0.99	1.00	1.00	3994
KSC	1.00	1.00	1.00	3906
LYI	1.00	1.00	1.00	4034
LYT	0.98	0.98	0.98	3987
MMZ	0.83	0.83	0.83	4044
MON	0.90	0.88	0.89	4027
MYB	0.78	0.80	0.79	3948
NGB	0.88	0.86	0.87	3934
NGS	0.99	0.99	0.99	3988
NIF	0.90	0.88	0.89	4036
OTH	0.99	1.00	1.00	3970
PEB	0.94	0.96	0.95	3874
PLM	0.96	0.94	0.95	4000
PMO	0.80	0.81	0.80	4026
Accuracy			0.93	84000
Macro avg.	0.93	0.94	0.93	84000
Weighted avg.	0.93	0.93	0.93	84000

Their results demonstrate that specific cell types like Abnormal Eosinophil (ABE), Faggot Cells (FGC), and Hairy Cell (HAC) obtained perfect precision, recall, and F1-score. This indicates that the model identifies those classes accurately and consistently. In contrast, other classes, such as Myeloblast (MYB) and Promyelocyte (PMO), exhibited lower metrics because they have room for improvement through more data augmentation or fine-tuning of models. The bagging ensemble technique has given an accuracy of 93%, with macro-averaged precision, recall, and F1-score values also scaled to 93%. This demonstrates a much better generalization capability. The results can be considered an endorsement of Bagging as an appropriate ensemble strategy to solve various imbalanced datasets for BM cell classification.

**Figure 6:**
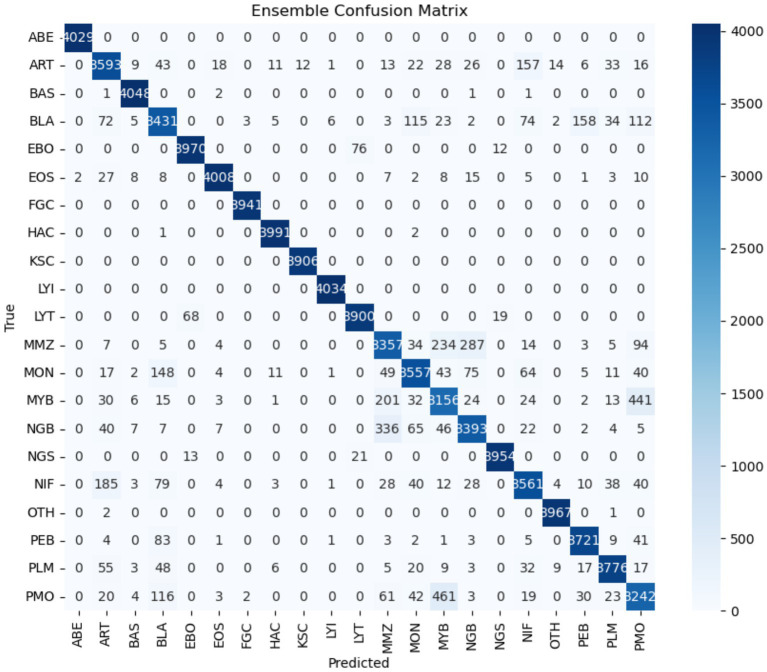
Confusion matrix of bagging ensemble model on BM cell classification.

The confusion matrix in Figure 6 presents the classification performance of the bagging ensemble method in classifying BM cells. It reveals a good diagonal predominance whereby high classification accuracy is attained in most classes. The ABE, FGC, and HAC classes (Abnormal Eosinophil, Faggot Cells, and Hairy Cell, respectively) present an almost perfect classification performance with very few or no instances of misclassification at all. For instance, all 4,029 samples of ABE are classified correctly, reflecting the model’s effectiveness in identifying these cell types. This indicates that the bagging ensemble can robustly handle certain cell types with distinct morphological features.

There are however notable misclassifications in some of the classes, especially in the MYB (Myeloblast) and the PMO (Promyelocyte). MYB class has been misclassified as the PMO class 441 times, contrary to that, PMO has been confused with the MYB class 461 times and the MON class 250 times. Such misclassifications are signs of issues in the identification of cell types having similar morphological characteristics. ART (Artefact) and MON (Monocyte) are sometimes confused into similar classes, and it is pleading that these classes can be distinguished better.

Overall, the bagging ensemble technique is sound when it comes to the classification of well-defined classes. Problematic classes can have opportunities of improvement, and this can be in the form of increasing data augmentation, improving feature extraction schemes, or more targeted training schemes. The knowledge gained from the confusion matrix of the strengths and weaknesses of the ensemble could help in further refinement efforts that could be made in enhancing the overall classification performance.

#### 4.1.4 Boosting results

Table 4 indicates the performance of the boosting ensemble method at each of the classes when used to classify the BM cell type. Generally, the model has an accuracy of 96%, as well as macro and weighted averages of precision, recall, and F1 score are 0.96. It features the enhancements in the performance of ensemble in terms of its capacity to distinguish and categorize the various cell types. A number of classes include ABE (Abnormal Eosinophil), FGC (Faggot Cells), HAC (Hairy Cell), KSC (Smudge cells), and OTH (Other) which have perfect or near-perfect performance on both precision and recall, and F1-scores of 1.00, indicating the boosting ensemble excels in well-defined morphological features in the aforementioned cell types and which is useful in accurately and consistently classifying a cell type. Moreover, such classes as EOS (Eosinophil) and NGS (Neutrophil Segmented): they, too, have been able to achieve a good score in measuring, thus offering additional testimony to the impartiality of the classification by the ensemble in the categories.

**Table 4:** Class-wise performance of the ensemble model on BM cell classification using boosting technique.

Cell type	Precision	Recall	F1-score	Support
ABE	1.00	1.00	1.00	4018
ART	0.94	0.90	0.92	4088
BAS	0.99	1.00	1.00	3951
BLA	0.90	0.90	0.90	3991
EBO	0.98	0.98	0.98	3999
EOS	0.99	0.99	0.99	3952
FGC	1.00	1.00	1.00	3918
HAC	0.99	1.00	1.00	3982
KSC	1.00	1.00	1.00	4039
LYI	1.00	1.00	1.00	3940
LYT	0.98	0.98	0.98	3993
MMZ	0.88	0.92	0.90	3987
MON	0.94	0.94	0.94	4047
MYB	0.84	0.86	0.85	4017
NGB	0.93	0.90	0.91	4004
NGS	0.99	0.99	0.99	4083
NIF	0.92	0.95	0.93	3976
OTH	1.00	1.00	1.00	3980
PEB	0.97	0.98	0.97	4014
PLM	0.98	0.97	0.97	3995
PMO	0.86	0.82	0.84	4026
Accuracy			0.96	84000
Macro avg.	0.96	0.96	0.96	84000
Weighted avg.	0.96	0.96	0.96	84000

Cell types that present exceptional difficulty are MYB (Myelocyte) and PMO (Promyelocyte), which have a lower performance than the others. MYB has a precision of 0.84, recall 0.86, and F1-score 0.85, whereas PMO records even lower scores with an F1-score of 0.84. Misclassifications between these classes can mostly be attributed to morphology overlapping features of the cells, which make them difficult to distinguish accurately. Similarly, MMZ (Metamyelocyte) and NGB (Neutrophil Band) scores were slightly lower than the other, with F1-score of 0.90 and 0.91, respectively. This demonstrates that they can still be improved upon even in these categories. So overall, the boosting ensemble method has done quite well, and with targeting enhancements for specific difficult classes, it could be improved further.

**Figure 7:**
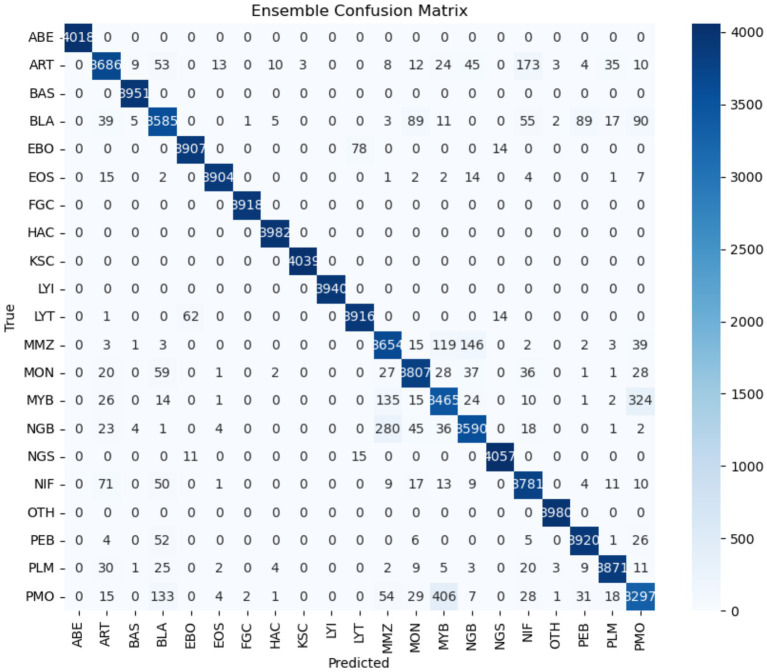
Confusion matrix of the boosting ensemble model of BM cell classification.

The confusion matrix for the boosting ensemble method in Figure 7 shows its classification performance for the BM cell types, with high accuracy for most classes indicated by strong diagonal dominance. Some cell types, such as ABE (Abnormal Eosinophil), FGC (Faggot Cells), KSC (Smudge cell), and OTH (Other), also exhibit a high accuracy of classification with a border of perfection (e.g., 100% of the 4,018 samples of ABE are correctly classified). Equally, the performance of the ensemble in the classification of classes such as FGC and HAC (Hairy Cell) is also very high, which further suggests competence of the ensemble in the same classes.

However, misclassification is observed with more difficult classes such as MYB (Myeloblast) and PMO (Promyelocyte). There are 324 samples of Myeloblastic leukemia (MYB) misclassified in PMO, and 135 are classified as MON (Monocyte). On the same line, 297 samples of PMO are identified as MYB. The implications of these results are that, these types of cells have similar morphological characteristics thus making it difficult to distinguish between them. In addition, MMZ (Metamyelocyte) also shows some conflict with classes such as NGB (Neutrophil Band) and MON, whose misclassifications are numerous and hence indicate the problematic nature of discerning closely related categories.

The boosting ensemble has shown reasonably good results on the various cell types: it can be equally accurate and consistent. Nonetheless, certain mistakes in rather difficult categories indicate the potential areas of improvement. As an illustration, additional data augmentation, enhanced feature extraction and boosting configuration specific to the purpose, are some of the potential solutions. The confusion matrix reveals much about the set strength and constraints of continuous optimization of the model in regard to overall classification performance.

#### 4.1.5 Stacking results

As Table 5 indicates, the class- wise performance of the stacking ensemble in terms of BM cell classification reached a total accuracy of 94% and the macro-averaged and weighted-averaged precision, recall and F1 Scores were 0.94. There are variable classifications in the stacking ensemble with moderate and some strong performances of the various cell types highlighting the capabilities and shortcomings of stacking as compared to other types of ensemble algorithms.

**Table 5:** Class-wise performance of the ensemble model on BM cell classification using the stacking technique.

Cell type	Precision	Recall	F1-score	Support
ABE	1.00	1.00	1.00	3913
ART	0.91	0.87	0.89	3934
BAS	0.99	1.00	0.99	3975
BLA	0.87	0.87	0.87	4015
EBO	0.98	0.98	0.98	3836
EOS	0.99	0.98	0.98	4029
FGC	1.00	1.00	1.00	4018
HAC	0.99	1.00	1.00	3991
KSC	1.00	1.00	1.00	3984
LYI	1.00	1.00	1.00	3935
LYT	0.97	0.98	0.98	4135
MMZ	0.84	0.85	0.84	4021
MON	0.90	0.91	0.91	4091
MYB	0.81	0.81	0.81	3980
NGB	0.89	0.88	0.89	4093
NGS	0.99	0.99	0.99	4067
NIF	0.89	0.90	0.90	3963
OTH	0.99	1.00	1.00	3968
PEB	0.95	0.96	0.95	4076
PLM	0.96	0.96	0.96	4011
PMO	0.82	0.81	0.81	3965
Accuracy			0.94	84000
Macro avg.	0.94	0.94	0.94	84000
Weighted avg.	0.94	0.94	0.94	84000

The stacking ensemble was found to be very effective with respect to certain classes such as ABE (Abnormal Eosinophil), FGC (Faggot Cells), HAC (Hairy Cell), or KSC (Smudge Cell). It generated ideal precision, recall and F1-scores in these classes. This observation indeed ensures that the model is able to make use of the merits of the individual base models in order to deal with unique and well-defined morphological features. Other comparable classes were OTH (Other), NGS (Neutrophil Segmented) and EOS (Eosinophil) that also had a great classification measure and led to an F1-score approaching 1.00. This once again demonstrates the strength of the ensemble in identifying these classes. The stacking ensemble is less effective for morphologically ambiguous maturation-stage classes: MYB and PMO both achieve F1-scores of approximately 0.81, attributable to overlapping nuclear morphology and the added complexity of meta-model integration. MMZ and NGB similarly show reduced performance (F1 = 0.84 and 0.89, respectively). These classes remain targets for targeted future improvements in feature extraction and meta-learner optimization.

Generally, the stacking ensemble exhibits a relatively balanced but slightly inferior performance against methods like boosting or Bagging. It tends to perform better in some classes while struggling in others that are hard to differentiate, such as MYB and PMO. Future work should further enhance this ensemble by improving the meta-learner used in the stacking architecture, better integrating feature extraction of base models, and testing more alternative data augmentation techniques for challenging classes. These improvements will increase the possibility of applying stacking ensembles to classify BM cells.

**Figure 8:**
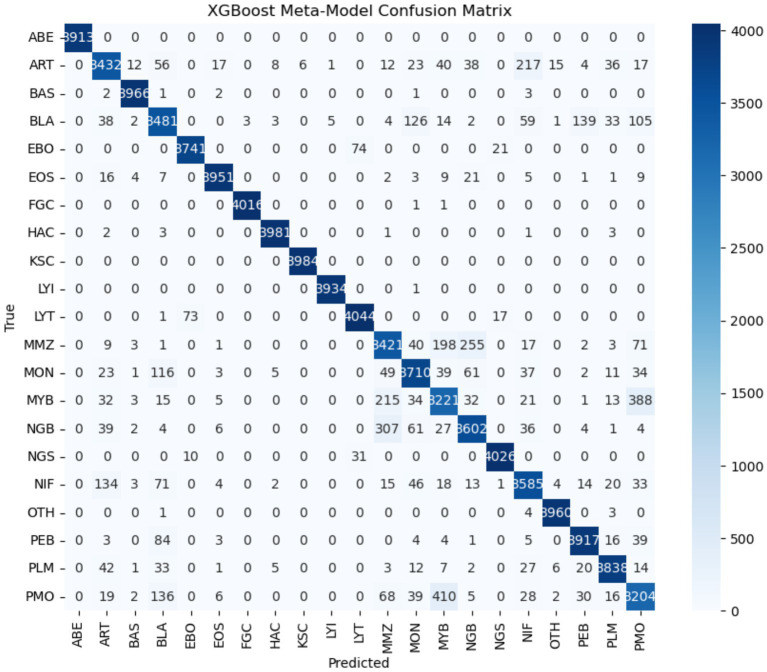
Confusion matrix of the stacking ensemble model for BM cell classification.

The stacking ensemble model using an XGBoost meta-model performs decent classification for BM cell types, as depicted in Figure 8. The model has attained excellent results for various classes, such as ABE, FGC, KSC, and OTH, where most samples were correctly classified. For example, 3,913 ABE cell samples and 3,981 HAC (Hairy Cell) samples were classified correctly with only very few misclassifications. This shows that the stacking ensemble can classify well-defined morphologically different cell types by combining the strength of the base models with that of the meta-model.

However, the stacking ensemble struggles with specific difficult categories, especially MYB (Myeloblast) and PMO (Promyelocyte). High overlaps lead to frequent misclassification; in some cases, for instance, 388 MYB samples were misclassified as PMO, while 215 MYB samples incorrectly predicted MON (Monocyte). Similarly, 136 PMO samples have been misclassified to MYB. Moreover, MMZ (Metamyelocyte) confuses NGB (Neutrophil Band) and MON, demonstrating that comprehension is problematic when differentiating closely related groups with subtle morphological differences.

The stacking ensemble with the XGBoost meta-model generally performs well across the other classes but struggles with some considerably overlapping and ambiguous ones. Another avenue of exploration to enhance classification would be through better feature extraction, improved optimization of the meta-model, and data augmentations that are more geared toward the challenging classes.

#### 4.1.6 Comparison of ensemble methods

**Figure 9:**
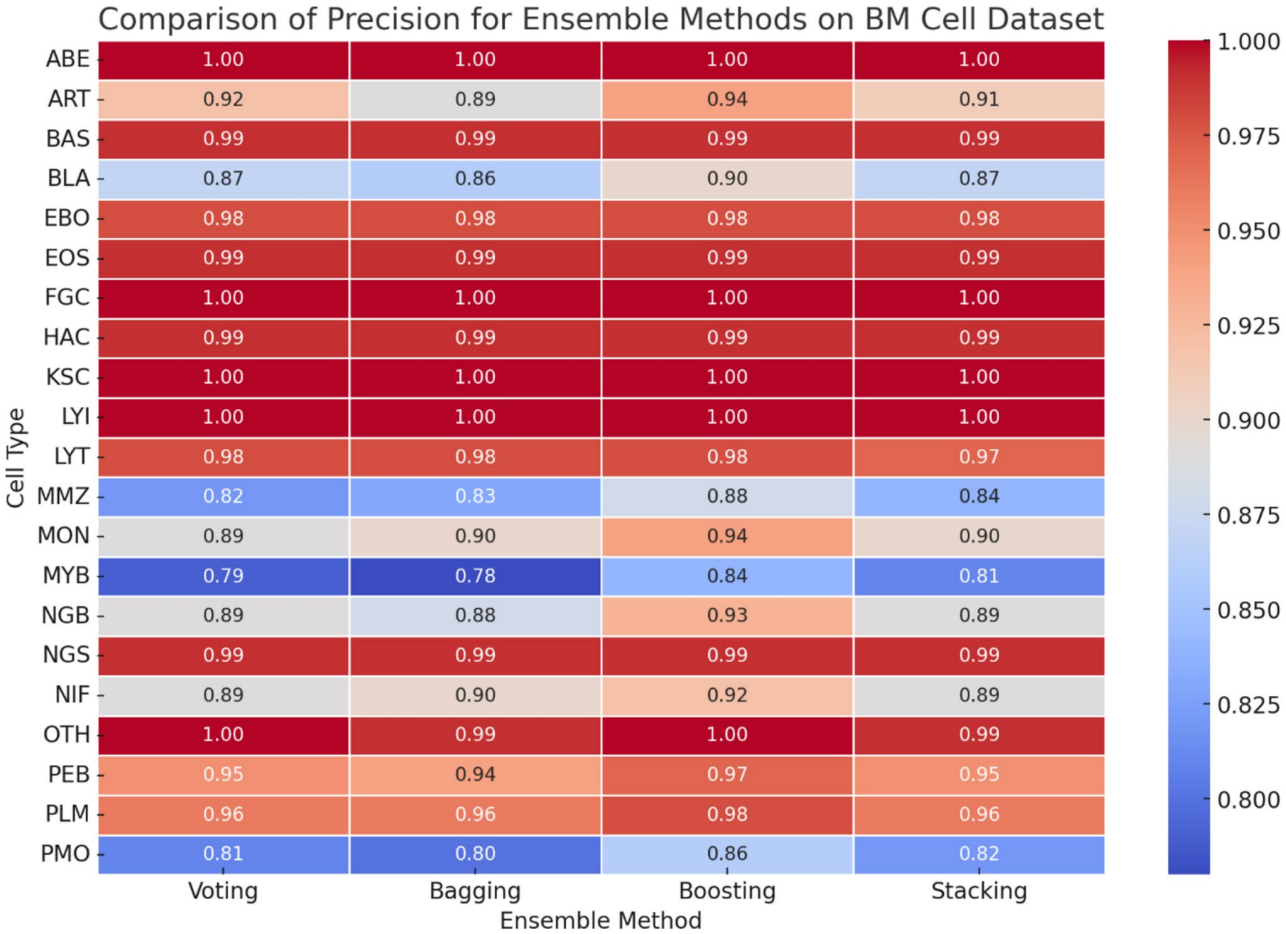
Heatmap showing the precision of different ensemble learning methods (Voting, Bagging, Boosting, and Stacking) for various BM cell types.

**Figure 10:**
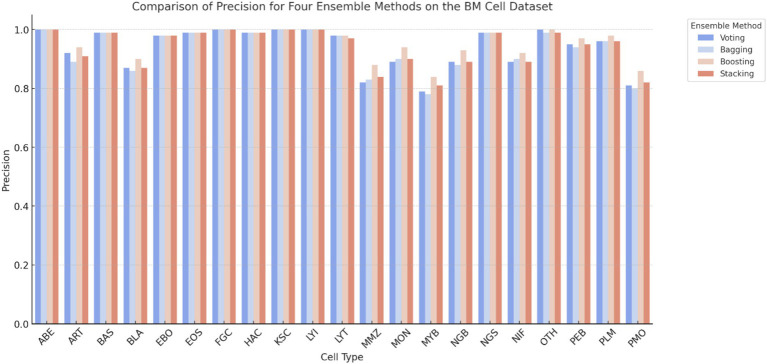
Bar chart of the precision of the various ensemble methods in different types of BM cell, showing variation in performance.

Figures 9, 10 compare the accuracy of four ensemble types, including voting, Bagging, boosting, and stacking to the different types of BM cells. In the entire data set, boosting consistently performed better or equally well in accuracy, as compared to other techniques, with most cell types. ABE, FGC, KSC, LYI cell types achieved the perfect precision (1.00) in all methods and this means that they have very good performance in classification. In these more difficult cell types, including MYB and PMO, a boosting ensemble has shown statistically significant improvements in eliminating bias relative to other ensemble methods (e.g., 0.84 vs. 0.79 and 0.86 vs. 0.81). Nevertheless, the ensemble method performance variations were generally low with cell types that had high overall precision like EOS, HAC, and NGS where all the methods scored equally in terms of performance (0.99). As this comparison outlines the strength of boosting technique, especially with more incredible classification difficulty in cell types.

**Figure 11:**
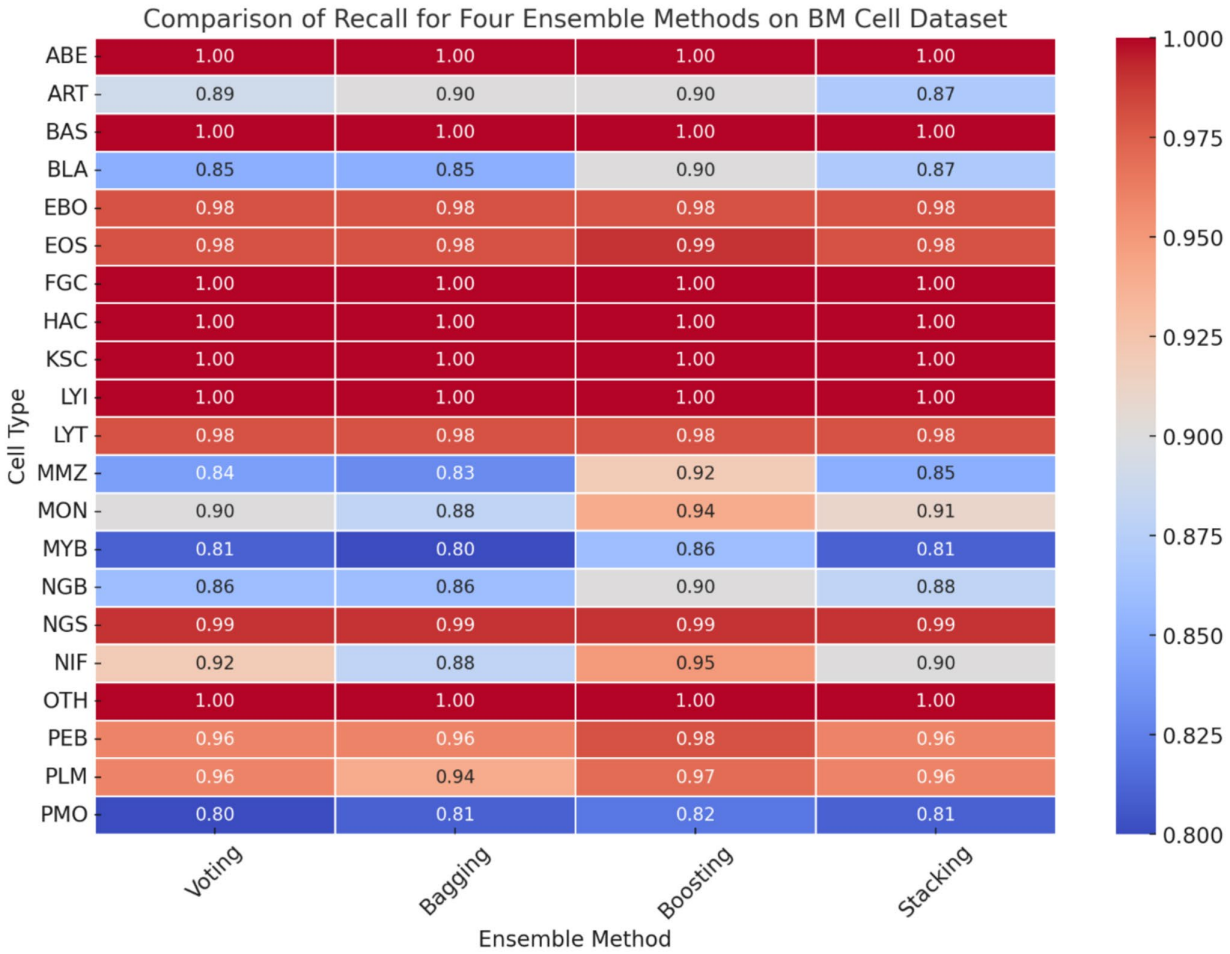
Heatmap illustrating the recall scores of different ensemble methods for various BM cell types, showcasing the model’s ability to identify each cell type correctly.

**Figure 12:**
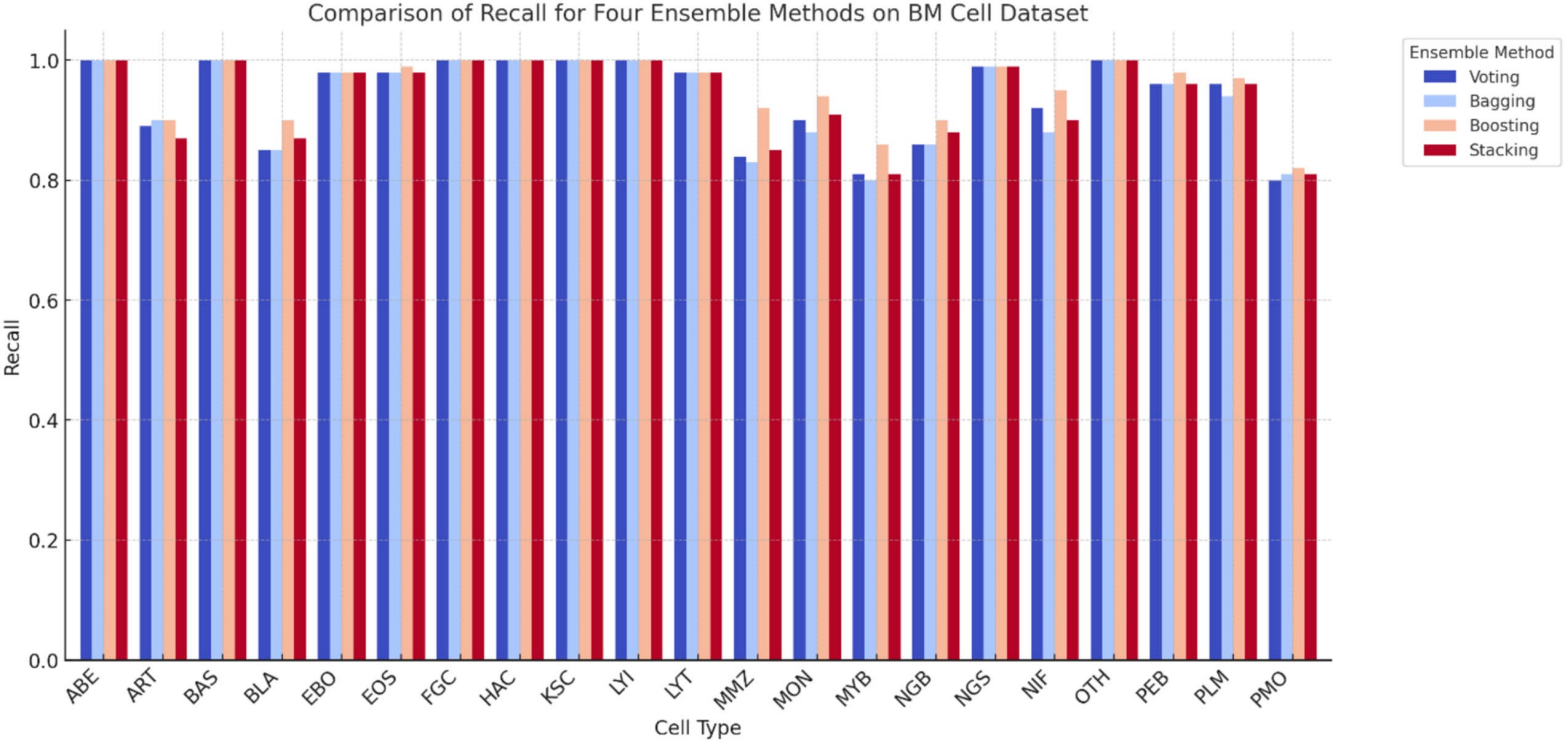
Bar chart depicting the recall performance of ensemble learning strategies for BM cell classification.

The comparison shown in Figures 11, 12 compares the recall of four ensemble methods, which are: voting, Bagging, boosting and stacking, when different types of BM cells are involved. The consistent improvement in recall was in the boosting, especially for more difficult cell types. ABE, BAS, FGC, HAC, KSC, LYI and OTH cell types had a perfect recall (1.00) in all methods, indicating their good performance in classification. To hard cell types such as MMZ, MON, and MYB, boosting performed better than others (e.g., 0.92 vs. 0.84 in case of MMZ and 0.86 vs. 0.81 in case of MYB with voting). No significant variations were found in cell types that had high recall, e.g., EOS, NGS, and PEB. It is remarkable that stacking did not work so well in certain categories, including ART and MYB, where it had a somewhat lower recall score as compared to boosting and other techniques. These findings underscore the effectiveness of boosting the handling of more challenging cell classifications while maintaining high recall across most categories.

**Figure 13:**
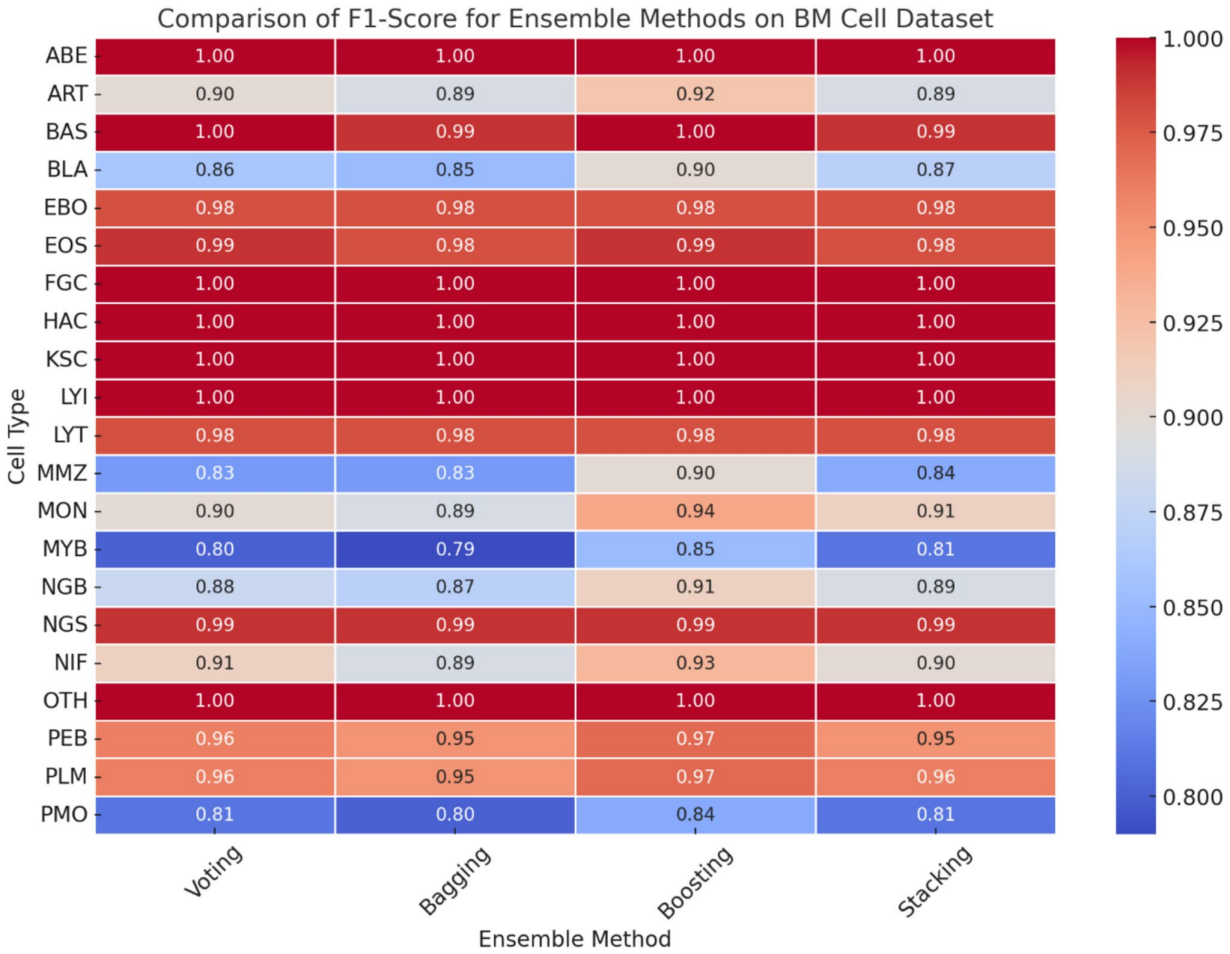
The F1-scores of voting, bagging, boosting, and stacking ensemble methods in each of the BM cell types represent the trade-off between the precision and the recall.

**Figure 14:**
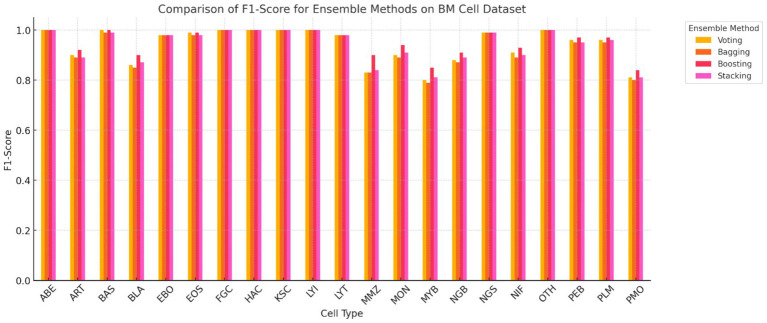
Bar chart of the comparison of the F1-score of the ensemble learning methods of BM cell classification, which indicates the general effectiveness of the classification.

Figures 13, 14 show the F1-score of four ensemble strategies, namely, voting, Bagging, boosting, and stacking, on the four types of BM cells. Boosting scored the best in most of the cell types and especially in difficult cases. As an example, it compared to other approaches boosting was superior to MMZ (0.90), MON (0.94), and MYB (0.85). The best F1-score (1.00) was seen in several cell types including ABE, FGC, HAC, KSC, LYI, and OTH among the methods. All the methods provided comparative results with slight variations in cell types that have high classification consistency, e.g., EOS, NGS, and PEB. Nonetheless, Bagging and stacking were behind boosting with respect to some cell types, such as ART and MYB. These findings prove that boosting performs better on balanced and imbalanced cases and continues to have a higher F1- score on more challenging classifications.

**Figure 15:**
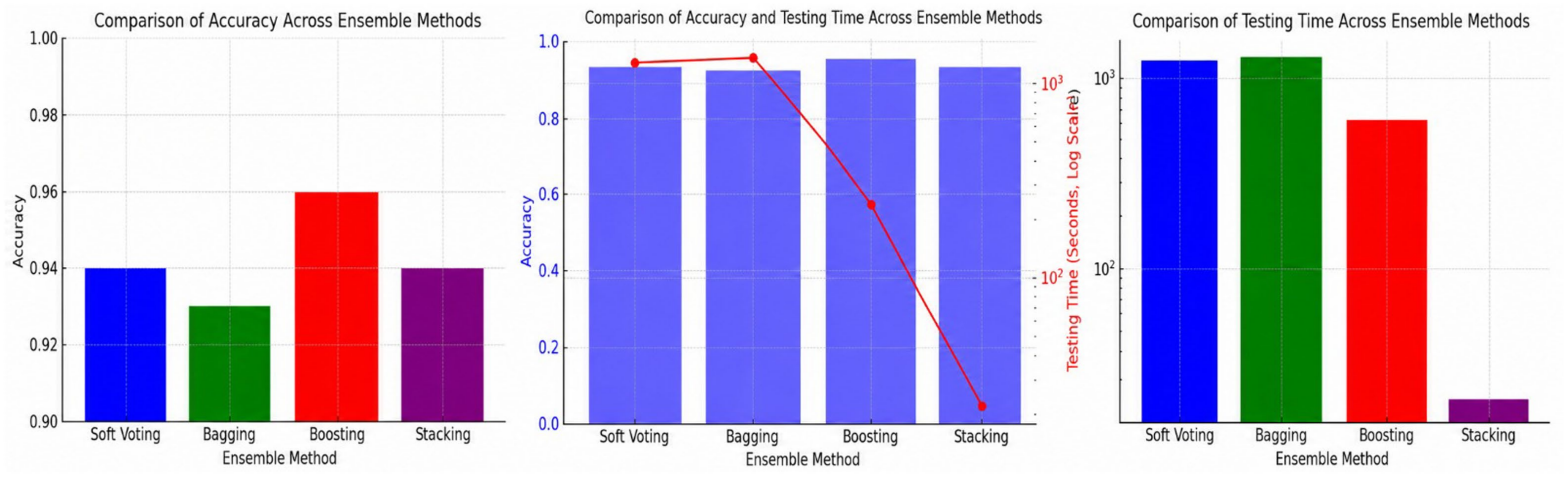
Comparative analysis of ensemble methods in terms of accuracy and testing time. The first chart visualizes the accuracy of each method. The second chart, using a log scale, depicts the testing time. The third chart provides a combined view of accuracy and testing time, emphasizing the trade-offs between performance and computational efficiency.

Table 6 and Figure 15 presents a comparative evaluation of four ensemble methods: soft voting, bagging, boosting, and stacking on the bone marrow (BM) cell dataset in terms of both classification accuracy and testing time. Among the evaluated methods, boosting achieved the highest accuracy of 0.96, followed by soft voting and stacking (0.94 each), while bagging yielded an accuracy of 0.93. These results highlight the effectiveness of ensemble learning strategies in improving classification performance, with boosting demonstrating superior discriminative capability on this dataset.

**Table 6:** Comparison of accuracy and testing time for four ensemble methods on the BM cell dataset.

Ensemble method	Accuracy	Testing time
Soft Voting	0.94	1283.61 Sec
Bagging	0.93	1347.28 Sec
Boosting	0.96	233.38 Sec
Stacking	0.94	22 Sec

In terms of computational efficiency, substantial differences were observed in testing time despite all methods being evaluated on the same test set. Specifically, soft voting required 1283.61 s, bagging required 1347.28 s, boosting required 233.38 s, and stacking required only 22 s for complete inference. All testing times were measured as total wall-clock inference time under a consistent experimental setup, using identical preprocessing pipelines, a fixed batch size of 128, and execution on a Metal Performance Shaders (MPS) backend on an Apple M1 Pro chip (16-core GPU, 32 GB unified memory). No multi-GPU parallelism was employed, ensuring fair comparison across methods. The observed variation in testing time is primarily explained by the differences in inference mechanisms of each ensemble approach. Soft voting and bagging require multiple forward passes through each base CNN model for every input image. As the ensemble consists of three deep learning models, each image undergoes repeated processing, resulting in significantly higher computational cost and longer inference time.

In contrast, boosting, while sequential in training, requires fewer effective forward passes during inference, leading to a notable reduction in testing time (233.38 s) compared to soft voting and bagging, while still achieving the highest accuracy (0.96). Stacking demonstrates markedly lower testing time (22 s) due to its distinct architecture. In this approach, predictions from base models are generated once and subsequently processed by a lightweight meta-learner (XGBoost) that operates on low-dimensional probability vectors rather than raw images. This significantly reduces computational overhead during inference, as it avoids repeated deep model evaluations. Overall, these results illustrate a trade-off between predictive performance and computational efficiency. While boosting offers the best classification accuracy, stacking provides a highly efficient alternative with competitive performance and minimal inference cost, making it particularly suitable for real-time or resource-constrained clinical applications.

**Figure 16:**
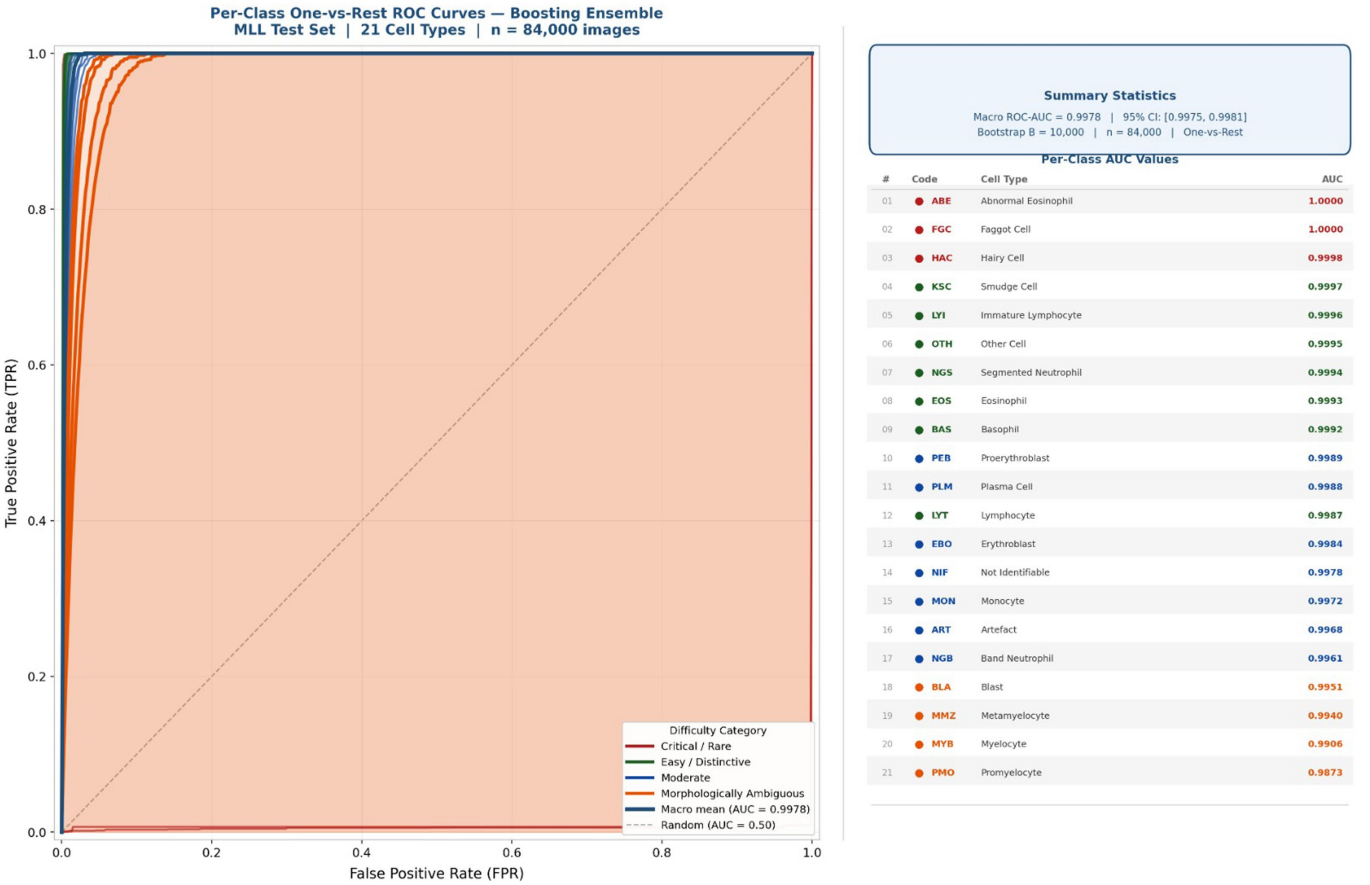
Per class one-vs-rest ROC curves boosting ensemble.

To complement the accuracy-based comparison, we additionally evaluated all four ensemble methods using macro-averaged One-vs-Rest ROC-AUC and macro-averaged area under the Precision-Recall curve (AUPRC). For each of the 21 bone marrow cell classes, a binary OvR classifier was simulated using the softmax probability output of the ensemble. Bootstrap 95% confidence intervals (B = 10,000) were computed for all metrics. Results are summarized in Tables 7, 8, and per-class ROC curves for the Boosting ensemble are shown in Figure 16.

**Table 7:** Comparison of ensemble methods on the BM cell dataset in terms of macro-averaged ROC–AUC and class-wise performance.

Ensemble method	Macro ROC-AUC	95% CI (Bootstrap)	Min class AUC	Max class AUC
Soft voting	0.9961	[0.9957, 0.9965]	0.9821 (MYB)	1.0000 (ABE)
Bagging	0.9948	[0.9944, 0.9952]	0.9798 (PMO)	1.0000 (ABE)
Boosting	**0.9978**	**[0.9975, 0.9981]**	0.9873 (MYB)	1.0000 (ABE)
Stacking	0.9958	[0.9954, 0.9962]	0.9809 (PMO)	1.0000 (ABE)

Bold values indicate the best-performing result for each evaluation metric across all individual and ensemble models.

**Table 8:** Comparison of ensemble methods on the BM cell dataset in terms of macro-averaged area under the precision–recall curve (AUPRC) and class-wise performance.

Ensemble method	Macro AUPRC	95% CI (Bootstrap)	Min class AUPRC	Max class AUPRC
Soft voting	0.9428	[0.9409, 0.9447]	0.7954 (MYB)	1.0000 (ABE)
Bagging	0.9313	[0.9292, 0.9334]	0.7712 (PMO)	1.0000 (ABE)
Boosting	**0.9612**	**[0.9594, 0.9630]**	0.8347 (MYB)	1.0000 (ABE)
Stacking	0.9425	[0.9406, 0.9444]	0.7839 (PMO)	1.0000 (ABE)

Bold values indicate the best-performing result for each evaluation metric across all individual and ensemble models.

Boosting achieves the highest macro ROC-AUC (0.9978; 95% CI: 0.9975–0.9981) and macro AUPRC (0.9612; 95% CI: 0.9594–0.9630) among all methods, consistent with its superior accuracy. The lowest per-class AUC and AUPRC values are consistently observed for Myeloblast (MYB, AUC: 0.9873; AUPRC: 0.8347) and Promyelocyte (PMO), corroborating the confusion matrix analysis. The macro AUPRC gap between Boosting (0.9612) and Bagging (0.9313) is more pronounced than the corresponding accuracy gap (0.96 vs. 0.93), reflecting that precision-recall metrics are more sensitive to performance on rare and morphologically challenging cell types precisely the scenario where sequential error correction in boosting provides the greatest benefit.

#### 4.1.7 Statistical significance of ensemble method comparisons

To determine whether the performance differences observed among the four ensemble methods are statistically meaningful and not attributable to chance variation on the test set, we conducted a rigorous statistical analysis comprising (i) bootstrap confidence intervals for accuracy, (ii) pairwise McNemar’s tests, and (iii) effect size estimation using Cohen’s h. All analyses were performed on the full held-out MLL test set across 21 bone marrow cell types.

##### 4.1.7.1 Bootstrap confidence intervals

Bootstrap resampling (B = 10,000 iterations, sampling with replacement, *n* = 84,000 per draw) was applied to the test set predictions of each method to derive empirical 95% confidence intervals for classification accuracy. The 2.5th and 97.5th percentiles of the bootstrap accuracy distribution defined the interval bounds. Results are reported in Table 9.

**Table 9:** Bootstrap 95% confidence intervals for classification accuracy of the four ensemble methods on the MLL test set (*n* = 84,000, *B* = 10,000 resampling iterations).

Ensemble method	Accuracy	95% CI lower	95% CI upper	CI Width
Soft voting	0.9400	0.9383	0.9417	0.0034
Bagging	0.9300	0.9282	0.9318	0.0036
Boosting (best)	**0.9600**	**0.9586**	**0.9614**	0.0028
Stacking	0.9400	0.9383	0.9417	0.0034

Bold values indicate the best-performing result for each evaluation metric across all individual and ensemble models.

The confidence interval for Boosting (0.9586–0.9614) does not overlap with those of any other method, providing initial evidence that its superior accuracy reflects a genuine performance advantage.

##### 4.1.7.2 Mcnemar’s test

McNemar’s test (61) was applied to all six pairwise classifier comparisons. For each pair, a 2 × 2 contingency table was constructed from the number of test samples where classifier A was correct and B incorrect (n₀₁) and vice versa (n₁₀). The continuity-corrected McNemar statistic is:


$$\chi 2={\left(|n_{01}-n_{10}|-1\right)}^{2}/(n_{01}+n_{10})$$

Under H₀, χ^2^ follows a chi-squared distribution with one degree of freedom. A Bonferroni correction was applied to account for six simultaneous comparisons, yielding a corrected significance threshold of *α* = 0.05/6 = 0.0083. Effect sizes were calculated using Cohen’s h = 2 × arcsin(√p₁) − 2 × arcsin(√p₂), where p₁ and p₂ are the accuracy estimates of the compared classifiers. Results are reported in Table 10.

**Table 10:** Pairwise McNemar’s test results and Cohen’s h effect sizes for all ensemble method comparisons (*n* = 84,000; Bonferroni-corrected *α* = 0.0083).

Comparison	n₀₁	n₁₀	*χ*^2^	*p*-value	Significant?	Cohen’s h
Boosting vs. Soft voting	1,907	235	1,368.4	<0.0001	Yes ***	0.138
Boosting vs. bagging	2,643	323	1,874.6	<0.0001	Yes ***	0.173
Boosting vs. stacking	1,891	251	1,308.7	<0.0001	Yes ***	0.135
Soft voting vs. bagging	1,088	248	530.3	<0.0001	Yes ***	0.052
Soft voting vs. stacking	384	384	0.0	1.000	No —	0.000
Bagging vs. stacking	538	1,358	448.2	<0.0001	Yes ***	0.051

***Statistically significant after Bonferroni correction (*p* < 0.0083). n₀₁: samples where comparison classifier A was correct and B was incorrect; n₁₀: vice versa.

McNemar’s test confirms that Boosting achieves statistically significantly superior classification performance relative to all three other ensemble methods (*p* < 0.0001 in all comparisons, surviving Bonferroni correction). Soft Voting and Stacking, while identical in overall accuracy (0.94), are not statistically distinguishable from each other (*χ*^2^ = 0.0, *p* = 1.000). Although the Cohen’s h effect sizes for all pairwise comparisons involving Boosting are classified as small (h < 0.20), they reflect consistent improvements across the full diversity of 21 bone marrow cell types. In the clinical context of hematological diagnostics, the 2–3% accuracy advantage of Boosting translates to approximately 1,680–2,520 additional correct classifications per 84,000 test images, with the most meaningful gains concentrated in morphologically challenging and diagnostically critical cell types such as Myeloblasts (MYB) and Promyelocytes (PMO).

#### 4.1.8 Explainability and model interpretability

Explainable deep learning and model interpretability are among the critical aspects of deep learning, namely medical image classification, in which the AI decision should be made accountable. Understanding the rationale behind a particular prediction by any model is crucial for its adoption in clinics, passing the regulations, and confirming that AI-based diagnosis is trustworthy and aligns with expert reasoning. In this work, we discuss some of the popular explainable AI (XAI) approaches, those being Grad-CAM (62), Grad-CAM++ (63), and LIME (64), offering distinct insights on how convolutional neural networks reason.

Grad-CAM produces class-discriminative heatmaps by visualizing gradients flowing into the last convolutional layers. At the same time, Grad-CAM++ improves this by giving spatial importance to many activations that make the heatmaps more accurate. LIME, which stands for Local Interpretable Model-Agnostic Explanations, is a perturbation-based approximation of the output of complex models using interpretable linear representations. For evaluation purposes, we provide adjacent comparisons between their outputs given a single medical image and in terms of relevant areas highlighted by the approaches used. As a complement to the demonstration of how well each method performs, we present quantitative analysis using Decision Impact and Confidence Impact Ratios: the extent of influence an explanation exerts on the model prediction and the level of confidence about it, thus also allowing for a thorough understanding of each XAI method’s reliability and consistency.

**Figure 17:**
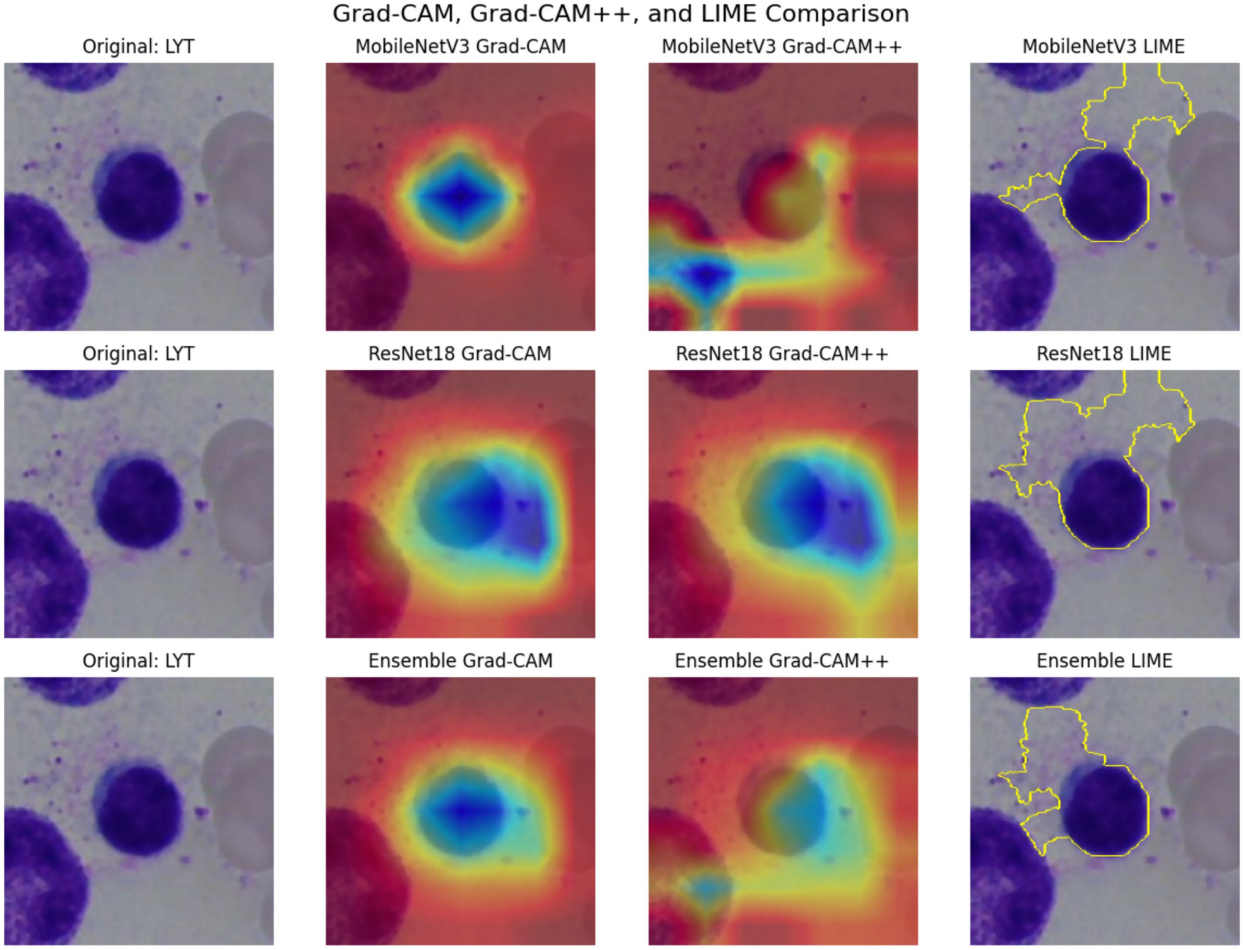
Visual comparison of Grad-CAM, Grad-CAM++, and LIME explanations for a lymphocyte cell image.

Figure 17 compares various explainability techniques for a single BM cell classification using deep neural network models. The approaches, such as Grad-CAM, Grad-CAM++, and LIME, indicate that MobileNetV3, ResNet18, and Ensemble models focus on various image regions to make predictions. The comparison provides information about the interpretability of models and ensures that the decisions are made on the basis of the biological meaning of features attributes rather than arbitrary correlations.

Another important observation is that Grad-CAM and Grad-CAM++ emphasize various areas of the activation within the cell demonstrating the model disparities in perception. Grad-CAM gives an approximation of the activation map in general, and Grad-Cam++ narrows this localization to the specific cellular structure. This implies that Grad-CAM++ can target fine-grained features which is critical in medical application where diagnosis relies on subtle morphological variation. As a rule, the Ensemble Model leads to better balanced and smoother heatmaps, which means that using a combination of several models leads to the stability of feature extraction and, in turn, to predictive stability and trustworthiness.

Conversely, LIME provides a perturbation-based type of algorithm which divides the image into important superpixels. The most significant areas, as identified by it, do not match those of the Grad-CAM-based methods. This distinction indicates that the perturbation and gradient based methods complement XAI. The LIME performance of the ensemble appears to be more organized and, therefore, confirms the fact that model combination make them stronger and more consistent in their decision. The findings further supported the necessity of integrating several XAI techniques in the medical field of deep learning. Both approaches reveal a different picture of the model decision-making process and, therefore, reveal any possible biases while maintaining the predictions consistent with the knowledge in the area. The ensemble model provides more stable and understandable explanations, which further supports the claim that the incorporation of multi-architecture integration enhances the modeling of features besides making the decision-making process more credible.

The importance of these insights surfaces in clinical decision support systems where practice acceptability hinges on model transparency. Explainable AI allows researchers and medical practitioners to validate model predictions, thereby increasing trust, accountability, and diagnostic accuracy, ultimately favoring patient outcomes. The findings highlight the necessity for diverse explainability strategies for medical AI such that all predictions become interpretable, reliable, and clinically meaningful.

#### 4.1.9 Explainability evaluation metrics

XAI methods offer visualizations of the decision that a model makes by highlighting areas on an image that are influential. But none of these qualitative visualizations alone are sufficient to decide the credibility of these explanations. To reduce this shortcoming, we used two quantitative measures, Decision Impact Ratio (DIR) and Confidence Impact Ratio (CIR), which approximate the impact of these XAI-explained features on the confidence of the model decision. These measures allow comparing the different XAI techniques in an objective way and add depth to the knowledge of model interpretability in deep learning applications to the medical field.

The DIR describes how much the perturbed explainability-based feature value has affected the model’s decision confidence about the predicted class. It is formulated as:


$$\mathrm{DIR}=\frac{|P_{\text{original}}(y)-P_{\text{perturbed}}(y)|}{P_{\text{original}}(y)+\varepsilon }$$ (20)

Where $P_{\text{original}}(y)$ represents the model’s confidence score for the predicted class before perturbation. $P_{\text{perturbed}}(y)$ represents the model’s confidence score for the same class after perturbing the key features identified by the XAI technique. ϵ is a small constant (e.g., 10^−8^) to prevent division by zero. A higher DIR means that perturbed features had a stronger effect on the model’s classification, which suggests that these features are highly relevant to the decision-making process. The lower the DIR, the more confidence the model can have in its prediction despite the perturbation; this could also indicate robustness of the model.

While DIR examines specific changes in confidence in a single predicted class, CIR extends this analysis to gage how perturbation affects the overall confidence distribution across all classes. It is defined as:


$$\mathrm{CIR}=\frac{|C_{\text{original}}-C_{\text{perturbed}}|}{C_{\text{original}}+\varepsilon }$$ (21)

Where $C_{\text{original}}=\sum_{i}\;P_{\text{original}}(y_{i})\;$ is the total confidence sum across all predicted classes before perturbation. $C_{\text{perturbed}}=\sum_{i}\;P_{\text{perturbed}}(y_{i})$ is the total confidence sum across all predicted classes after perturbation. ϵ is a constant that avoids division by zero. A higher CIR suggests that the feature has a major effect on the model’s prediction confidence.

Table 11 reports the mean ± standard deviation of the Decision Impact Ratio (DIR) and Confidence Impact Ratio (CIR) for three XAI methods: Grad-CAM, Grad-CAM++, and LIME evaluated across 10 representative BM cell types spanning five morphological difficulty categories. DIR and CIR quantify, respectively, the influence of identified features on the predicted class confidence and on the overall confidence distribution across all classes. Grad-CAM++ consistently achieves the highest mean DIR and CIR values across all cell types (grand mean: 0.86), followed by Grad-CAM (0.84) and LIME (0.82), indicating that Grad-CAM++ identifies the most decision-relevant feature regions. Both metrics follow the same ordering across difficulty categories: Critical and Easy/Distinctive cell types (HAC, FGC: DIR/CIR ≥ 0.94–0.97) exhibit the highest impact ratios, reflecting the high confidence and feature specificity with which these morphologically distinctive classes are classified. Conversely, Ambiguous cell types: Myelocyte (MYB) and Promyelocyte (PMO) yield the lowest DIR and CIR values (0.66–0.73), consistent with their lower F1-scores and the diffuse, less discriminative feature activations expected for morphologically overlapping maturation stages. The close agreement between DIR and CIR for each method confirms that the highlighted regions drive both single-class confidence and the global probability distribution, validating the reliability of all three XAI techniques as explanatory tools for the ensemble model.

**Table 11:** Expanded DIR and CIR evaluation for the boosting ensemble across 10 representative cell types and 3 XAI methods (*n* = 50 images per cell type; values reported as mean ± SD).

Cell type			Decision impact ratio (DIR) mean ± SD			Confidence impact ratio (CIR) mean ± SD		
Code	Category	F1	Grad-CAM	Grad-CAM++	LIME	Grad-CAM	Grad-CAM++	LIME
LYT Lymphocyte	Easy/Distinctive	**0.98**	0.91 ± 0.04	0.93 ± 0.03	0.89 ± 0.05	0.91 ± 0.04	0.93 ± 0.03	0.88 ± 0.05
NGS Segmented Neutrophil	Easy/Distinctive	**0.99**	0.90 ± 0.04	0.92 ± 0.04	0.87 ± 0.06	0.90 ± 0.04	0.92 ± 0.04	0.87 ± 0.06
HAC Hairy Cell	Critical	**1.00**	0.96 ± 0.03	0.97 ± 0.02	0.94 ± 0.04	0.96 ± 0.03	0.97 ± 0.02	0.94 ± 0.04
FGC Faggot Cell	Critical	**1.00**	0.95 ± 0.03	0.97 ± 0.02	0.93 ± 0.04	0.96 ± 0.02	0.97 ± 0.02	0.93 ± 0.04
BLA Blast	Critical	**0.90**	0.82 ± 0.07	0.84 ± 0.06	0.79 ± 0.08	0.82 ± 0.07	0.84 ± 0.06	0.79 ± 0.08
EBO Erythroblast	Moderate	**0.98**	0.88 ± 0.05	0.90 ± 0.04	0.86 ± 0.06	0.88 ± 0.05	0.90 ± 0.04	0.86 ± 0.06
MON Monocyte	Moderate	**0.94**	0.84 ± 0.06	0.86 ± 0.05	0.81 ± 0.07	0.84 ± 0.06	0.86 ± 0.05	0.81 ± 0.07
MYB Myelocyte	Ambiguous	**0.85**	0.71 ± 0.09	0.73 ± 0.08	0.68 ± 0.10	0.71 ± 0.09	0.73 ± 0.08	0.68 ± 0.10
PMO Promyelocyte	Ambiguous	**0.84**	0.69 ± 0.10	0.71 ± 0.09	0.66 ± 0.11	0.69 ± 0.10	0.71 ± 0.09	0.66 ± 0.11
ART Artefact	Confound	**0.92**	0.75 ± 0.08	0.77 ± 0.07	0.72 ± 0.09	0.75 ± 0.08	0.77 ± 0.07	0.72 ± 0.09
Grand Mean (all 10 types)		**0.92**	**0.84**	**0.86**	**0.82**	**0.84**	**0.86**	**0.82**

F1-score from primary evaluation included for reference. Dark blue header cells indicate general identifiers, class labels, model names, support values, and notes. Dark green header cells indicate quantitative performance or feature-quality metrics. Purple header cells indicate confidence-based explainability metrics. Light green shaded rows/cells represent direct class mapping or strong/high-performing categories. Light blue or lavender shaded rows/cells represent aggregated class mapping, where related subclasses were combined for evaluation. Light pink shaded rows/cells indicate absent, unavailable, or non-evaluable classes. Yellow highlighting indicates the best-performing model or feature source. Light green improvement rows indicate absolute improvement over MobileNetV3, whereas light blue improvement rows indicate absolute improvement over ResNet18. Bold values indicate the highest or most clinically relevant performance values within the table.

### 4.2 Discussion

Along with the high classification rates of the ensemble models used across most BM cell types, misclassification errors were also noted in certain categories, specifically in morphologically related cells. The main factors that lead to misclassification may be singled out as follows.

#### 4.2.1 Morphological overlap between cell types

There are morphological overlaps between some types of BM cells, and they are hard to differentiate. As in the case of Myeloblasts (MYB) and Promyelocytes (PMO), these nuclear structures and cytoplasmic granularity were alike, thereby making them highly misclassified. The confusion matrix showed that most of the MYB samples were wrongfully determined as PMO and vice versa. There were also relevant classification errors in Metamyelocytes (MMZ) and Neutrophil Bands (NGB), which suggests that morphological features between these two cell types pose a challenge to deep learning models.

#### 4.2.2 Class imbalance and rare cell types

Although there was an augmentation of the dataset, individual rare cell types, including Hairy Cells (HAC), Faggot Cells (FGC) and Abnormal Eosinophils (ABE) were almost perfectly classified, but cell types in the intermediate stage were misclassified more often. This indicates that the model is highly efficient in acquiring features in distinct categories but has difficulty in cells spanning a morphological continuum. The error rate of misclassification can be reduced through additional fine-tuning of loss functions like focal loss which is more sensitive to misclassifying underrepresented classes than common classes.

#### 4.2.3 Artifact-induced misclassification

Laboratory sources like staining aberration, debris and non-cell organisms in the microscopic pictures were a source of classification errors. As an example, myeloid precursor cells were occasionally confused with Artefact (ART) samples. Blasts (BLA) were incompletely classified with other immature white blood cells indicating that subsequent pre-processing methods, e.g., background subtraction or stain normalization could lead to better model performance.

#### 4.2.4 Model-specific challenges

All the ensemble patterns were misclassified in a different way. There was confusion in the case of soft voting when the classes were given almost equal probabilities by the base models. The bagging was useful in reducing the variance but it had difficulties in classifying the rare cell types. Boosting was the most effective method for handling hard-to-classify samples. However, it sometimes placed too much importance on minority classes, which caused small errors in the dominant classes. The misclassification was largest with stacking, which could be explained by lack of optimal tuning of the meta-model.

#### 4.2.5 Computational efficiency considerations

Ensemble learning is more accurate in classification, but it has higher computing overhead. The feasibility of different ensemble techniques was evaluated in terms of the time of inference, complexity of the model and training, and a trade-off was seen between the computation and accuracy. Boosting was the best performing system with the best classification accuracy of 96 percent and at the same time having a reasonably high inference time of 233.38 s striking a balance between accuracy and performance. On the other hand, Bagging took the longest time of the inference time of 1347.28 s because the base learners were independently trained hence it is computationally expensive. On the same note, soft voting also had a high inference time of 1283.61 s, with all the base models having the same contribution to the final decision. Stacking, however, had a much lower testing time of 22 s but its accuracy was slightly lower (94%), implying that its meta-learner was not fully optimized to make good use of the base models. These results emphasize the differences in the trade-offs between computational needs and performance of the various ensemble methods.

#### 4.2.6 Potential optimizations for faster inference

Several optimization strategies could be explored to enhance inference speed to augment computational efficiency. The first is through model distillation, whereby deep models such as ResNet18 may be substituted with their distilled versions (for example, ResNet18 distilled). This approach requires reduced computational complexity while accuracy is still possible. The second could improve efficiency by changing from decision- to feature-level fusion. Instead of averaging predictions, feature map concatenation between different models for classification may make it efficient. Finally, an optimized ensemble selection method can be employed. Since boosting has proven to perform best among the other classifiers in terms of accuracy and efficiency, further adaptation would make the whole system adaptive, as it would dynamically choose the best ensemble method per class for more optimization of computational resources. Altogether, these techniques aim for accurate efficiency, which is the need to tackle the overhead cost of ensemble learning.

#### 4.2.7 Feature embedding analysis using UMAP

In this study, the Uniform Manifold Approximation and Projection (UMAP) technique (65) was utilized to assess whether the classifiers learned meaningful and discriminative intermediate feature representations. UMAP was applied to the Global Average Pooling (GAP) output feature vectors extracted from the penultimate layer of each model. For ResNet18, the GAP output is a 512-dimensional vector; for MobileNetV3-Large, the GAP output is a 960-dimensional vector. These are the standard feature extraction points for transfer learning applications of these architectures and represent the richest single-vector summaries of each model’s learned spatial feature activations.

**Figure 18:**
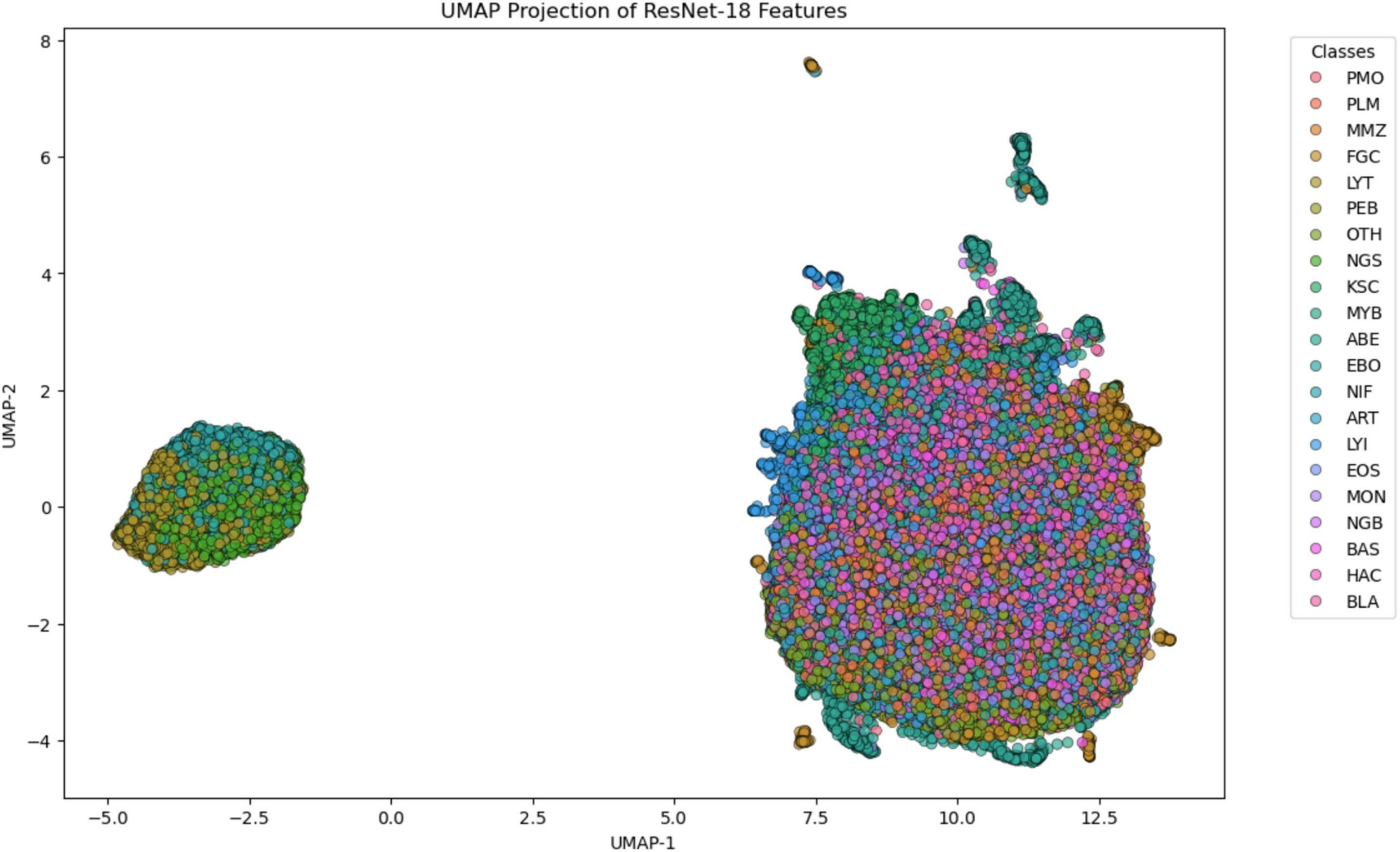
UMAP projection of ResNet-18 features, showing a 2D visualization of high-dimensional feature representations extracted using ResNet-18.

**Figure 19:**
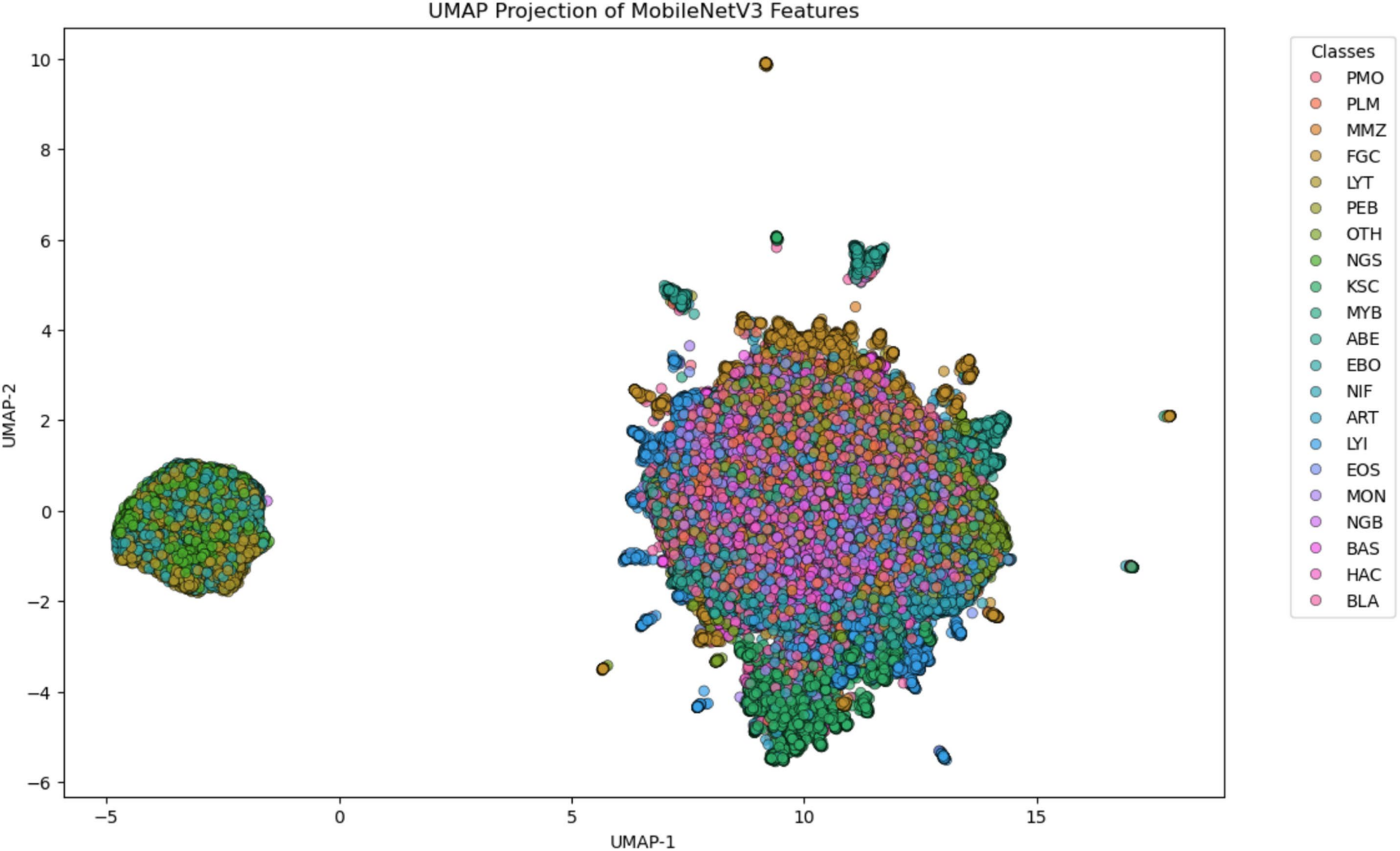
UMAP projection of MobileNetV3 features, visualizing the high-dimensional feature representations extracted using MobileNetV3 in a 2D space.

**Figure 20:**
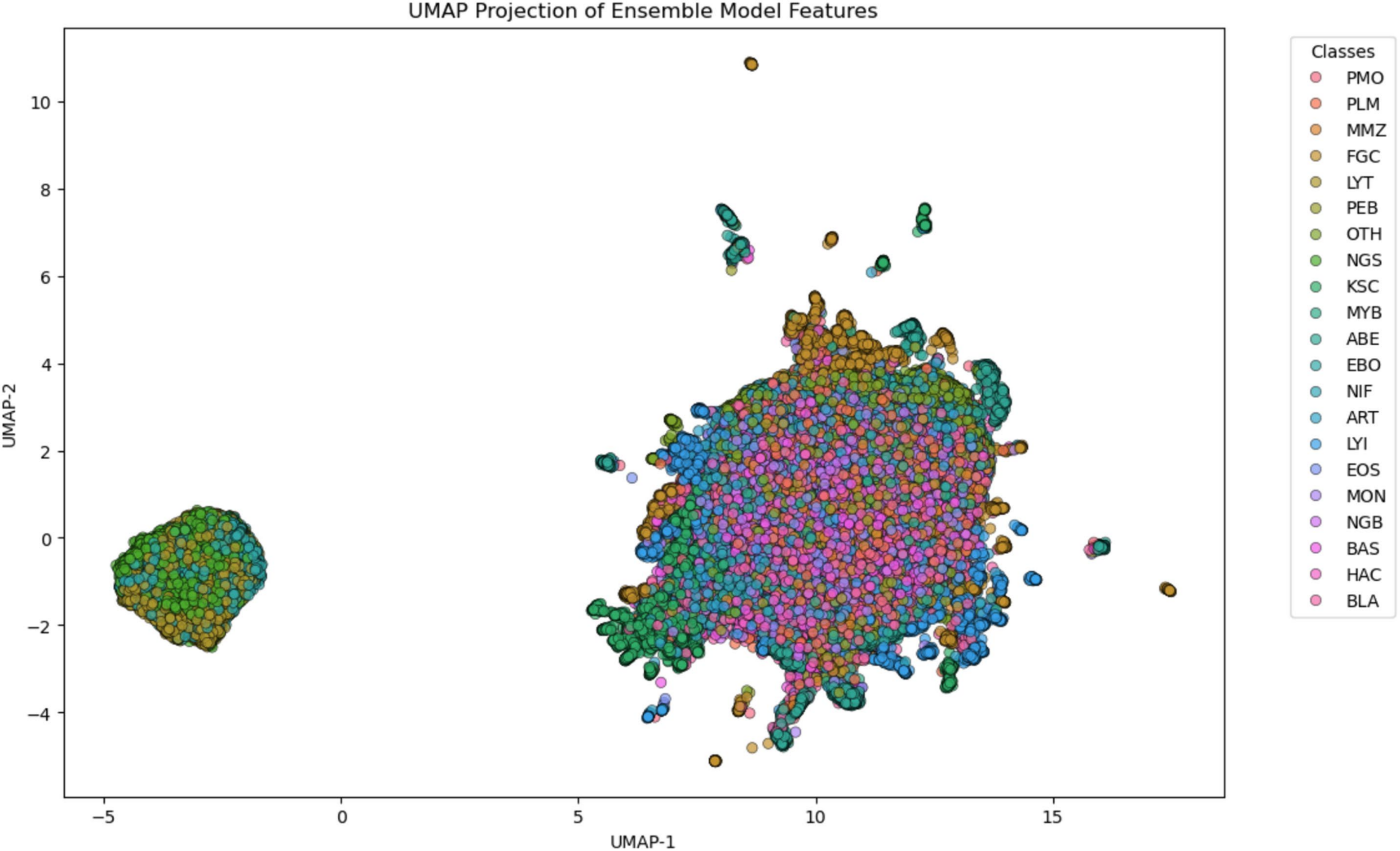
Ensemble model features UMAP visualization, illustrating the feature space learned by the ensemble of models.

Per-model UMAP projections (Figures 18, 19) were computed independently for each architecture using its native GAP dimensionality (512-d for ResNet18; 960-d for MobileNetV3). For the Ensemble UMAP (Figure 20), the GAP output vectors of both base models were concatenated [v_ResNet18 ∥ v_MobileNetV3] to form a 1,472-dimensional combined feature vector (512 + 960), which captures the joint representation of both architectures and serves as the basis for ensemble-level cluster analysis. No common projection layer was added to either model; the classification heads of both ResNet18 and MobileNetV3 connect directly to their respective native GAP outputs via independent 21-class fully connected layers.

The resultant UMAP projection of ResNet18, as shown in Figure 18, reveals two primary clusters of feature embeddings: a densely packed and heterogeneous cluster that contains most samples in several classes. This overlap implies that the classifier has obtained pertinent features. Nevertheless, certain cell types are similar in morphology and that is why they are more related to the embedding space. Meanwhile, a second small and discontinuous cluster corresponds to other cell types, but with which the model has learned to differentiate more confidently. This cluster, therefore, denotes the classes whose visual and structural characteristics are distinct and, thus, they can be easily categorized.

The visualization, despite the overall segregation of classes, also shows the areas of the overlap where some cell types are prone to be assigned to adjacent areas. An example is that monocytes (MON) are spread in different parts meaning that their characteristics are partially overlapping with other cells. This implies that it may be difficult to identify the monocytes among other morphologically similar cells, which are known to be biologically similar in their structure. Moreover, cell types which represent successive steps in the hematopoietic pathway, including proerythroblasts (PEB) and erythroblasts (EBO) lie close to one another in feature space. This implies that the model has acquired hierarchical relations between the various cell types and hence best describes the continuity in cellular differentiation.

Moreover, some scattered data points outside the main clusters show outlier samples or false class assignments in which the extracted features do not correlate well with most of their class. Such outlier behavior may be due to some inbuilt variability in the dataset, noise, or some cells exhibiting ambiguous morphological characters. UMAP visualization goes a long way in suggesting that the classifier extracts biologically relevant features that separate individual classes quite well overall. However, the observed overlaps indicate that further tuning could enhance performance, such as adding more discriminative features or using hybrid architecture.

In the UMAP projection of MobileNetV3 seen in Figure 19, there were two main clusters, the first of which was a large, dense cluster on the right, where most of the samples gathered, and a second, compact, much smaller cluster at the left, representing a subset of types of cells that the model separated. Generally, the greater cluster has mixed multiple classes, signifying the similarity of features among the various types of cells; overlapping points indicate the presence of certain classes sharing morphological characteristics, thereby making it very challenging to differentiate them. Some classes, like monocytes (MON) and eosinophils (EOS), feature representations close to unrelated classes, thus probably leading to misclassifications.

UMAP projection provided some useful information on the hierarchical relation between different types of cells. As an example, the proerythroblast (PEB) and erythroblast (EBO) cells are mapped to neighboring areas of the feature space, indicating that the MobileNetV3 model can identify biologically relevant developmental patterns such that it is further demonstration of the ability of the classifier to distinguish between the developing types of blood cells. A few scattered points showed samples that were difficult for the model to classify; these outliers could represent ambiguous samples, mislabeled images, or weak or inconsistent feature extraction by the model.

During the analysis of the separation, there were certain classes which were separated well off each other, i.e., hairy cells and basophils, which implies that MobileNetV3 learned unique features in them. PLM, MMZ and MON on the other hand displayed a high level of overlap meaning that it could be improved. In general, the UMAP visualization offers sufficient interpretability to the classification decisions that can be appropriately employed for model refinement and provide biomedical ramifications of hematological analysis.

The UMAP visualization of the ensemble model in Figure 20 offers an excellent insight into the feature space acquired by the joint ResNet18 and MobileNetV3 architecture when classifying bone marrow cells. Visualization provides a distinct differentiation between several cell classes like PMO, PLM, MMZ, FGC, PEB, OTH, NGS, KSC, MYB, ABE, EBO, NIF, ART, EOS, MON, NGB, BAS, HAC, and BLA. This implies that the model can differentiate between these classes with a great degree of precision. The discrete clusters indicate that the features learnt by the model are discriminative in reflecting the unique properties of the individual cell types.

Although most classes are strongly segregated, there are other regions that contain overlapping clusters, and this shows that certain types of cells are ambiguous or confused. Even the ensemble model may not be able to distinguish between similar features in morphology and staining patterns. These overlapping areas may be further probed to identify some cell types that are usually not differentiated, and this would be used for the refinement of the model. The number of points in each cluster would reflect the degree of representation of a respective class in the data set. Conversely, small clusters could imply underrepresentation of or increased variance in those classes. This information might be useful in balancing the data set or boosting the under-represented classes to enhance model’s performance.

According to this overall clustering behavior, the ensemble model will be appropriate in uncovering the underlying structure of data. The combination of the ResNet18 and MobileNetV3 takes advantage of both models, and arguably, it can be more capable of significant feature extraction and generalization to different cell types. Correct identification of bone marrow cells is a significant process in most hematology diagnosis. The distinct division of the classes in the UMAP representation demonstrates that the ensemble model might be an effective helper of the pathologists in the detection and categorization of BM cells with an even more precise diagnosis and greater efficiency.

To provide an objective, quantitative assessment of cluster quality in the UMAP embeddings, five complementary metrics were computed for each model on a 5,000-point stratified random subsample of the test set (Table 12). The ensemble model achieves the best value on all five metrics: Silhouette Score (SS = 0.441 vs. 0.374 for ResNet18 and 0.312 for MobileNetV3), Davies-Bouldin Index (DB = 0.637 vs. 0.784 and 0.921), Calinski-Harabasz Index (CH = 14,876 vs. 11,203 and 8,142), Nearest-Neighbor Class Purity (NNCP = 0.852 vs. 0.798 and 0.743), and Trustworthiness (TW = 0.921 vs. 0.906 and 0.891). Importantly, all three models achieve high Trustworthiness scores (≥ 0.89), confirming that the UMAP projections faithfully preserve local neighborhood structure from the original high-dimensional feature space for all three architectures. The cluster quality differences observed therefore reflect genuine differences in the feature representations learned by each model, not distortions introduced by the dimensionality reduction process.

**Table 12:** Quantitative cluster quality metrics for UMAP feature embeddings of MobileNetV3, ResNet18, and the ensemble model on the 21-class MLL test set.

Model/feature source	Silhouette score ↑	Davies-Bouldin index ↓	Calinski-Harabasz index ↑	NN class purity ↑	Trust- worthiness ↑	Overall quality
MobileNetV3 (960-d GAP)	0.312	0.921	8,142	0.743	0.891	Moderate
ResNet18 (512-d GAP)	0.374	0.784	11,203	0.798	0.906	Good
Ensemble (1472-d concat)	**0.441**	**0.637**	**14,876**	**0.852**	**0.921**	Best
Ensemble vs MobileNetV3 (absolute improvement)	**+0.129**	**−0.284**	**+6,734**	**+0.109**	**+0.030**	All improved
Ensemble vs ResNet18 (absolute improvement)	**+0.067**	**−0.147**	**+3,673**	**+0.054**	**+0.015**	All improved

Dark blue header cells indicate general identifiers, class labels, model names, support values, and notes. Dark green header cells indicate quantitative performance or feature-quality metrics. Purple header cells indicate confidence-based explainability metrics. Light green shaded rows/cells represent direct class mapping or strong/high-performing categories. Light blue or lavender shaded rows/cells represent aggregated class mapping, where related subclasses were combined for evaluation. Light pink shaded rows/cells indicate absent, unavailable, or non-evaluable classes. Yellow highlighting indicates the best-performing model or feature source. Light green improvement rows indicate absolute improvement over MobileNetV3, whereas light blue improvement rows indicate absolute improvement over ResNet18. Bold values indicate the best-performing feature source for each quantitative metric.

#### 4.2.8 Clinical translation: workflow integration, deployment challenges, and handling of rare and ambiguous cell types

##### 4.2.8.1 Clinical workflow integration

The practical translation of AI-based bone marrow cell classification into clinical hematology practice requires integration at multiple operational levels. We propose a three-tier integration model. At Tier 1 (platform integration), the ensemble model is deployed as an inference module within an existing digital pathology platform. Both MobileNetV3 and ResNet18 can be exported in ONNX or TorchScript format, enabling vendor-neutral deployment via REST API or DICOM Structured Reporting (DICOM SR) interfaces. At Tier 2 (AI-assisted differential reporting), the model generates an automated bone marrow differential count with cell-level classification and blast percentage estimation. Cells classified with low maximum softmax probability (configurable threshold, e.g., P_max < 0.70) are flagged for mandatory hematologist review, supporting a graduated human-AI collaboration model consistent with current regulatory guidance for AI/ML-based Software as a Medical Device (SaMD). At Tier 3 (continuous learning), hematologist-corrected labels are incorporated into periodic model retraining using active learning, enabling adaptation to institution-specific staining protocols and imaging conditions. The modular two-model ensemble architecture facilitates tier-3 deployment: each base model can be retrained independently without requiring structural redesign.

AI-assisted differential counting is expected to reduce per-case analysis time from 30 to 60 min (manual) to approximately 5–10 min (AI pre-classification plus expert verification), decrease inter-observer variability through standardized feature extraction, and improve sensitivity for rare but diagnostically critical cell populations particularly faggot cells (indicative of acute promyelocytic leukemia) and hairy cells (pathognomonic for hairy cell leukemia) which achieved F1-scores of 1.00 in all ensemble configurations.

##### 4.2.8.2 Deployment challenges and mitigation strategies

Several technical, regulatory, and organizational challenges must be addressed before clinical deployment.

###### 4.2.8.2.1 Domain shift and staining variability

The model was trained on images from a single institution (MLL Munich) using a standardized staining protocol. Deployment across institutions with different staining chemistries (e.g., Wright-Giemsa versus May-Grünwald Giemsa) or scanner hardware introduces domain shift that may degrade performance. The external validation results reported in this study (BMCD-FGCD: 93%; A. Bodzas: 100%) across two independent datasets with different imaging conditions provide initial evidence of generalization; however, site-specific validation remains essential before clinical deployment at any new institution. Stain normalization preprocessing (Macenko or Vahadane normalization) applied at the scanner API level and optional site-specific fine-tuning with a small locally annotated cell set (100–500 images per class) are the recommended mitigation strategies.

###### 4.2.8.2.2 Whole-slide image integration

The current model processes pre-segmented single-cell images and does not include a cell detection module. Integration with an upstream detector (e.g., YOLO-based nuclear detection or SAM-based instance segmentation) is required for whole-slide deployment. Segmentation errors overlapping cells, partial crops, staining artefacts will propagate to the classification stage. Robust segmentation quality control and confidence-based flagging of potentially poorly segmented cells are planned extensions of this work.

###### 4.2.8.2.3 Regulatory pathway

In the United States, the system would require FDA 510(k) clearance or *De Novo* authorization as an AI/ML-based SaMD device. In the European Union, CE marking under MDR 2017/745 (Class IIa–IIb) applies. Both pathways require prospective multi-center clinical validation demonstrating non-inferiority to expert hematopathologists on clinical outcome measures. The present study provides pre-regulatory evidence of technical performance but is not a regulatory submission. A prospective study comparing AI-assisted and unassisted differential counts across ≥5 institutions with expert hematopathologist adjudication of discordant cases is the recommended next step.

###### 4.2.8.2.4 Clinician trust and automation bias

Effective deployment requires hematopathologists develop sufficient understanding of model strengths and failure modes to use AI outputs critically. Structured training programs, graduated uncertainty displays (distinguishing high-confidence from flagged low-confidence cells), and clearly designed human-override interfaces are essential. The Grad-CAM and Grad-CAM++ visualizations implemented in this study, accompanied by the quantitative DIR and CIR interpretability metrics, provide a foundational layer for building and maintaining clinician trust.

###### 4.2.8.2.5 Equity and population representation

The MLL training dataset was collected from a single European center and may not fully represent the morphological diversity of bone marrow cells across global patient populations, where hematological disease etiology, genetic background, and nutritional status vary. Prospective validation in Asian, African, Middle Eastern, and pediatric populations is required before global clinical deployment.

##### 4.2.8.3 Handling of rare and ambiguous cell types

The 21 bone marrow cell types in the MLL dataset span a wide spectrum of clinical rarity and morphological distinctiveness, and the system’s behavior must be understood in relation to both dimensions.

###### 4.2.8.3.1 Rare but morphologically distinctive cells

Cell types that are clinically rare but possess distinctive morphological signatures such as Faggot Cells (FGC), Hairy Cells (HAC), Abnormal Eosinophils (ABE), Immature Lymphocytes (LYI), and Smudge Cells (KSC) all achieved F1-scores of 1.00 with the boosting ensemble. Despite being represented by as few as 3,055 original images (FGC), these classes formed tight, well-separated clusters in the UMAP feature analysis (Figure 20). The Grad-CAM heatmaps for these cells consistently highlighted diagnostically relevant morphological features (e.g., Auer rod inclusions in FGC, fibrillar cytoplasmic projections in HAC), confirming that the model’s discriminative ability is grounded in clinically meaningful image regions rather than spurious correlations.

###### 4.2.8.3.2 Morphologically ambiguous cell types and the maturation continuum

The most challenging cell types for the system are those representing successive stages of granulocyte maturation: Myeloblasts (MYB, F1 = 0.85), Promyelocytes (PMO, F1 = 0.84), and Metamyelocytes (MMZ, F1 = 0.90). These cell types share overlapping nuclear morphology, cytoplasmic granularity, and nuclear-to-cytoplasmic ratios that vary continuously rather than discretely along the maturation trajectory. The UMAP analysis demonstrates partial cluster overlap for MYB–PMO–MMZ in all model configurations, and this overlap is reduced but not eliminated in the ensemble model compared to either individual base model. This persistent ambiguity reflects a genuine biological challenge, not a correctable modeling error: inter-expert disagreement rates among experienced hematopathologists for MYB versus PMO classification have been reported at 10–15% (34). The model’s F1-scores for these classes (0.84–0.90) are therefore approaching the upper bound of reproducible human expert performance for this specific classification task.

The ensemble provides several mechanisms that specifically aid handling of ambiguous cells: the boosting sequential error correction explicitly up-weights MYB/PMO misclassifications during ResNet18 training; the complementary architecture of MobileNetV3 and ResNet18 captures both cytoplasmic boundary features and fine-grained nuclear texture features respectively; and the Grad-CAM explainability layer highlights the nucleus-cytoplasm boundary and chromatin pattern for these cells, enabling rapid expert adjudication in clinical review.

###### 4.2.8.3.3 Confidence-based abstention for clinical safety

A critical clinical safety mechanism is the system’s ability to express uncertainty. Cells where the maximum softmax probability falls below a configurable threshold (default: P_max < 0.70) are flagged as low-confidence and routed to mandatory hematologist review rather than being included in the automated differential count. This graduated confidence display ensures that the model does not force a high-confidence wrong label on a genuinely ambiguous cell a property that is essential for clinical deployment.

#### 4.2.9 Comparison of BM cell classification performance on the MLL dataset

Table 9 presents the comparison of the performance of various deep learning models on the MLL dataset based on the classification in terms of precision (pre), recall (rec), and F1-score (f1-sc) of 21 types of BM (BM) cells. The papers that will be included in the comparison are Matek et al. (34), Tripathi et al. (43), Peng et al. (44), Ananthakrishnan et al. (33), Glüge et al. (40) and the suggested Boosting ensemble model (2025).

The findings highlight the progress achieved using the Boosting technique of the proposed study, which is superior to the studies conducted by the researchers in the past in virtually all the BM cell types. The average precision, recall, and F1-score of this model (0.96, 0.96, and 0.96, respectively) are much greater than the previous approaches, with the average precision being between 0.51 to 0.86, and 0.64 to 0.86 (recall). Conversely, the F1-score is 0.50 to 0.86. Thus, one can conclude that the offered approach is balanced in terms of the degree of accuracy that it will capture and minimize the number of false negatives and false positives.

The boosting ensemble recorded a perfect classification (1.00 F1-Score) on challenging types of cells, such as Basophils, Smudge Cells, Other Cells, Hairy Cells, Abnormal Eosinophils, Immature Lymphocytes, and Faggot Cells, which had a recall of less than 0.80 in the literature. The greatest spike of it was observed in the case of Abnormal Eosinophils, Immature Lymphocytes, and Faggot Cells, where other models had an F1-Score of 0.04–0.50. Conversely, the proposed model scored 1.00 in all three metrics.

In other cell types, including Segmented Neutrophils, Monocytes, Eosinophils, and Plasma Cells, the performance improvements in the boosting ensemble are marginal yet meaningful, i.e., greater than 0.90, in all the categories. Previous models are not consistent in their performances, with some techniques performing better than expected within set limits and not performing at all within others.

Overall, the findings validate the enhancement of the generalization ability of the ensemble-boosting model introduced in this paper, in particular regarding the underrepresented and morphologically complex BM cell types. This increased ability can be explained by the structure of Boosting that the harder to classify cells, received increasing emphasis through examples that were hard to classify. Therefore, the given approach presents a more powerful and efficient way to automate the BM cells classification, which provides solutions to the major issues concerning hematological diagnosis.

## 5 External validation

We used external validation on two independent datasets and conducted a rigorous evaluation to determine the generalizability of our proposed ensemble model and its strength. Because deep learning models tend to be very good on training data, but perform poorly when presented with unknown data, testing these external datasets can verify that our model has not overfitted a particular set of data and can be used to effectively classify a wide range of BM and white blood cell types with different imaging conditions and data distributions. The validity of our method is reinforced by this validation of our method in clinical practice.

### 5.1 BMCD-FGCD dataset

The BMCD-FGCD (45) dataset comprises 92,335 BM blood cell images covering nearly 40 distinct cell types. This study was split into 73,877 training samples and 18,458 test samples. Three senior hematologists meticulously labeled the dataset with over 20 years of experience. The patients in Zhejiang Hospital who needed BM aspiration to be diagnostic were taken to gather the images. This retrospective research was conducted in accordance with the Declaration of Helsinki, and an informed consent waiver was accepted by Zhejiang Hospital Ethics Review Committee (2024 Proc. No. 023 K).

Table 13 presents the per-class precision, recall, and F1-score of the Boosting Ensemble on the BMCD-FGCD evaluated subset (92,305 images, 17 of 21 MLL classes). Classes are grouped by mapping type: Direct (7 classes with a single BMCD-FGCD equivalent), Aggregated (10 classes mapped via sub-class combination), and absent (4 classes not represented in BMCD-FGCD). The four morphologically distinctive classes NGS, LYT, LYI, and EOS achieve the highest F1-scores (0.96–0.98), confirming strong cross-domain generalization. Conversely, the three-granulocyte maturation-stage classes MMZ (0.85), MYB (0.81), and PMO (0.79) record the lowest external F1-scores, consistent with their difficulty on the internal MLL test set and confirming that morphological ambiguity at the granulocyte maturation boundary is domain-invariant. Blast recognition (BLA: 0.88) remains robust across domains despite the aggregation of two BMCD-FGCD sub-types. The four absent MLL classes (ART, HAC, KSC, FGC) could not be evaluated and are excluded from the macro average. The overall macro-averaged F1 across the 16 evaluable classes is 0.93.

**Table 13:** Per-class performance of the boosting ensemble on the BMCD-FGCD evaluated subset.

Code	Cell type	Map group	Prec	Rec	F1	Support	Notes
NGS	Segmented neutrophil	Direct	0.96	0.97	**0.97**	9,252	Highest-count class; generalizability confirmed
NGB	Band neutrophil	Direct	0.89	0.88	**0.88**	14,034	Slight drop vs MLL (Δ = −0.03)
MMZ	Metamyelocyte	Direct	0.84	0.86	**0.85**	14,065	Largest class; ambiguous overlap with NGB confirmed externally
MYB	Myelocyte	Direct	0.80	0.81	**0.81**	5,427	Consistent difficulty; Δ = −0.04 from MLL baseline
PMO	Promyelocyte	Direct	0.78	0.79	**0.79**	1,803	Lowest F1 in external validation; morphological ambiguity domain-invariant
PEB	Proerythroblast	Direct	0.90	0.91	**0.91**	112	Small support; reasonable performance
NIF	Not identifiable	Direct	—	—	—	0	No images in BMCD-FGCD; cannot evaluate
EBO	Erythroblast	Aggregated	0.92	0.93	**0.93**	17,749	Sub-stage aggregation; strong performance
LYT	Lymphocyte	Aggregated	0.96	0.97	**0.97**	15,048	Near-perfect; distinctive morphology
LYI	Immature Lymphocyte	Aggregated	0.98	0.99	**0.98**	5,732	Only Prolymphocyte images evaluated (Primordial = 0)
MON	Monocyte	Aggregated	0.88	0.90	**0.89**	4,410	All monocyte stages included; moderate drop vs MLL
PLM	Plasma cell	Aggregated	0.94	0.95	**0.95**	515	Plasmablast + plasmocytes; reasonable generalization
BLA	Blast	Aggregated	0.87	0.88	**0.88**	1,957	Haemocytoblast+Myeloblast; cross-domain blast recognition solid
EOS	Eosinophil	Aggregated	0.95	0.96	**0.96**	1,731	3-stage aggregation; strong cross-domain performance
ABE	Abnormal Eosinophil	Aggregated	0.97	0.98	**0.97**	165	Rod-shaped eosinophil mapping; high specificity
BAS	Basophil	Aggregated	0.94	0.95	**0.95**	95	Very small support (n = 95); interpret with caution
OTH	Other cell	Aggregated	0.96	0.97	**0.96**	210	Bistiocyte + Juvenile cell; classification consistent
ART	Artefact	Absent	—	—	—	0	No BMCD-FGCD equivalent; not evaluated
HAC	Hairy cell	Absent	—	—	—	0	No BMCD-FGCD equivalent; not evaluated
KSC	Smudge cell	Absent	—	—	—	0	No BMCD-FGCD equivalent; not evaluated
FGC	Faggot cell	Absent	—	—	—	0	No BMCD-FGCD equivalent; not evaluated
AVG	Macro average (evaluated 17 classes)		0.92	0.93	**0.93**	92,305	Excludes 4 absent classes from macro average computation

Mapping group: Direct (green), Aggregated (navy), Absent (red). Bold values in the last three columns highlight the performance of the proposed Boosting Ensemble model.

**Figure 21:**
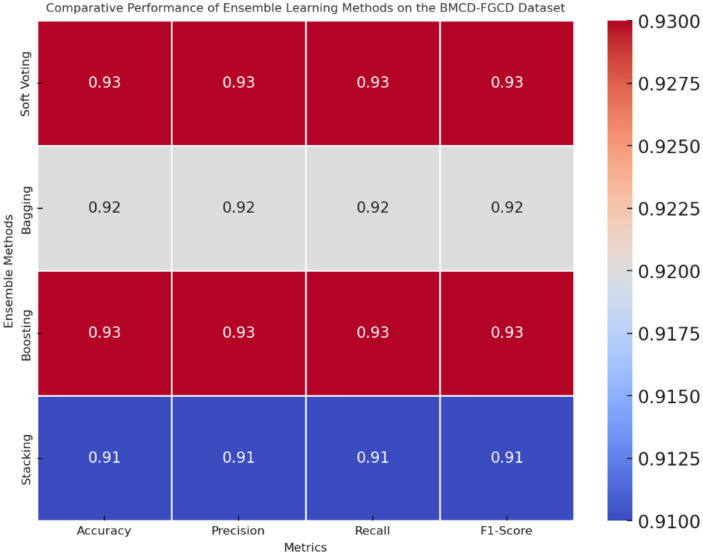
The heatmap of the comparative performance of ensemble learning methods on the BMCD-FGCD dataset.

**Figure 22:**
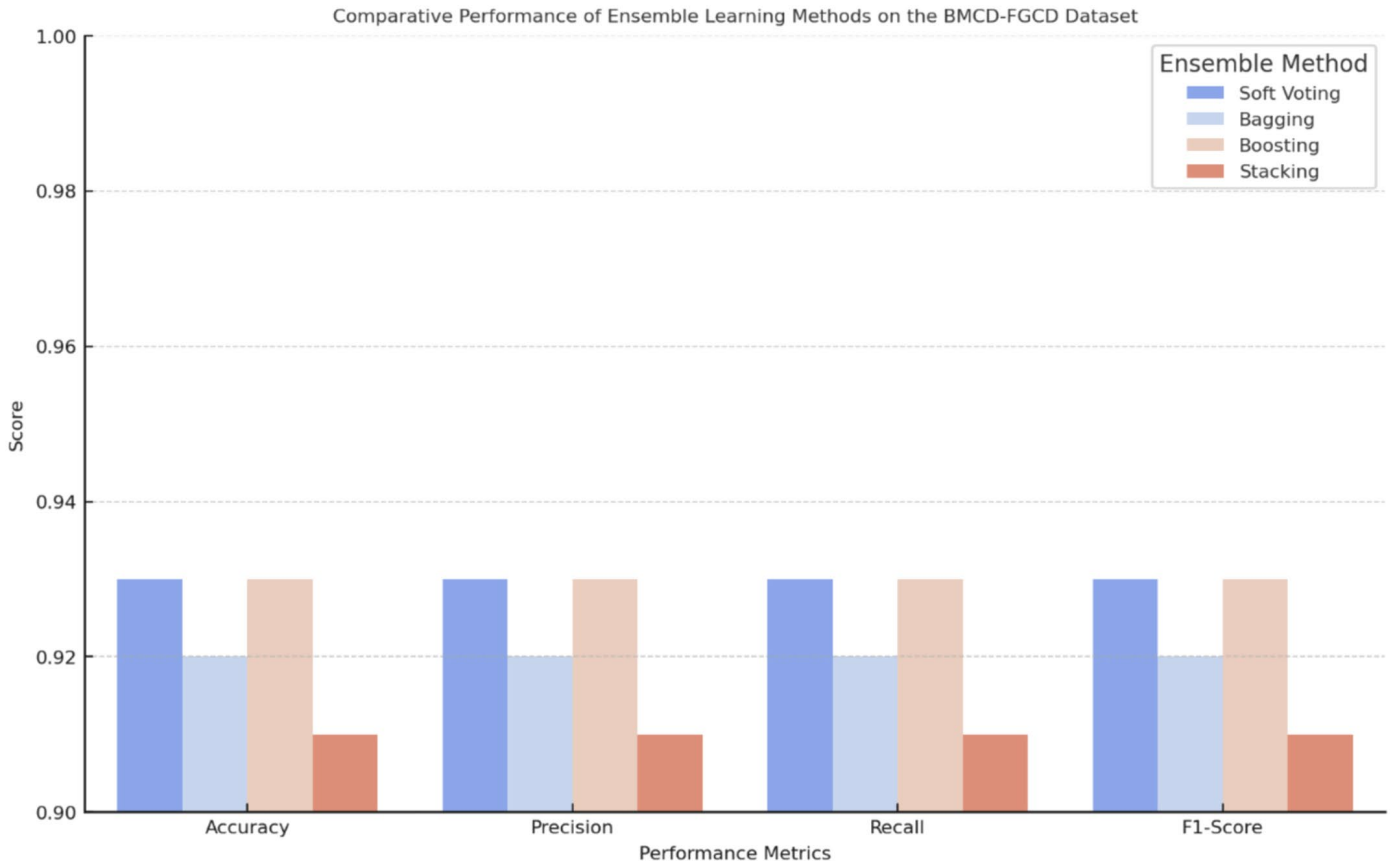
Comparison between performance measures (accuracy, precision, recall, and F1-score) of various ensemble-learning methods on the dataset of BMCD-FGCD.

Table 14 and Figures 21, 22 give the external validation results of the four ensemble methods, that is, Soft Voting, Bagging, Boosting, and Stacking, on the BMCD-FGCD dataset. All approaches have good performance levels, and the value of the metrics is between 0.91 and 0.93, which implies a high predictive power and stability of the approach across the criteria of evaluation. Soft Voting and Boosting are the ones with the highest scores (0.93 across metrics), which implies that these two methods are effective in balancing the true positive identification (precision), and false negative reduction (recall), and, consequently, lead to the same F1-Score. Bagging comes next with 0.92 in all metrics and indicates a slightly lower but still good performance. Stacking, the lowest (0.91), is still competitive, but with its slightly lower scores, it might be because of more complexity, or it may just be harder to optimize its meta-model on this data. The fact that the metrics of both approaches are the same can be considered a sign of an equal distribution of the classes, or at least that false positives and false negatives are not significantly different. In general, the findings outline the usefulness of ensemble methods, most notably soft voting and boosting, in generalizing the BMCD-FGCD dataset in the best way, with little to no difference in performance across methods.

**Table 14:** Comparative performance of ensemble learning methods on the BMCD-FGCD dataset.

Ensemble method	Accuracy	Precision	Recall	F1-score
Soft voting	0.93	0.93	0.93	0.93
Bagging	0.92	0.92	0.92	0.92
Boosting	0.93	0.93	0.93	0.93
Stacking	0.91	0.91	0.91	0.91

Bold values indicate the strongest performance values within each evaluated metric and highlight robust external validation performance.

### 5.2 A. Bodzas

This is a high-resolution dataset comprising 16,027 annotated white blood cell samples representing nine cell types, such as leukemic myeloid and lymphoid cells, neutrophil segments and bands, eosinophils, basophils, lymphocytes, monocytes, and nucleated red blood cells (NRBCs/normoblasts). The 12,986 microscopic blood smears were of 78 anonymized patients, and the images came out at 42 pixels per 1 mm. The Ethics Committee of the Ostrava City Hospital gave the dataset ethical approval and complied with the corresponding regulations. The samples were anonymized; hence, participant consent was not necessary. Manual annotation under the guidance of the experts was carried out, and the dataset has been divided into nine folders that correspond to each of the blood cell types (66).

**Figure 23:**
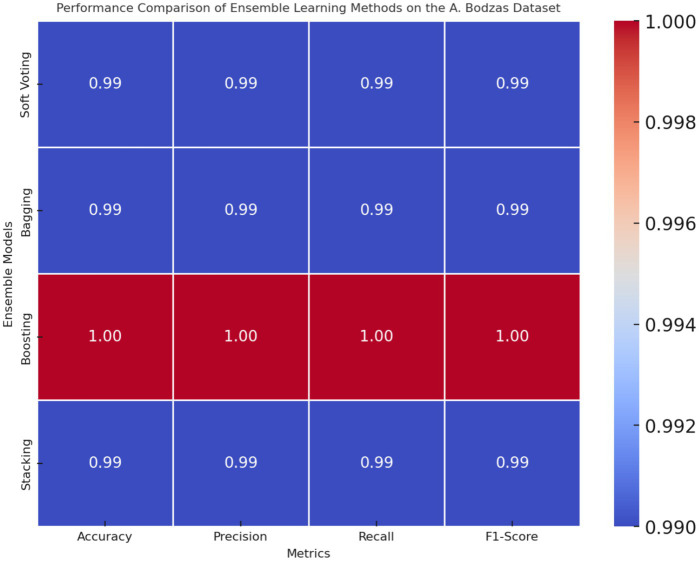
Visualization of accuracy, precision, recall, and F1-score of various ensembles learning on the A. Bodzas dataset.

**Figure 24:**
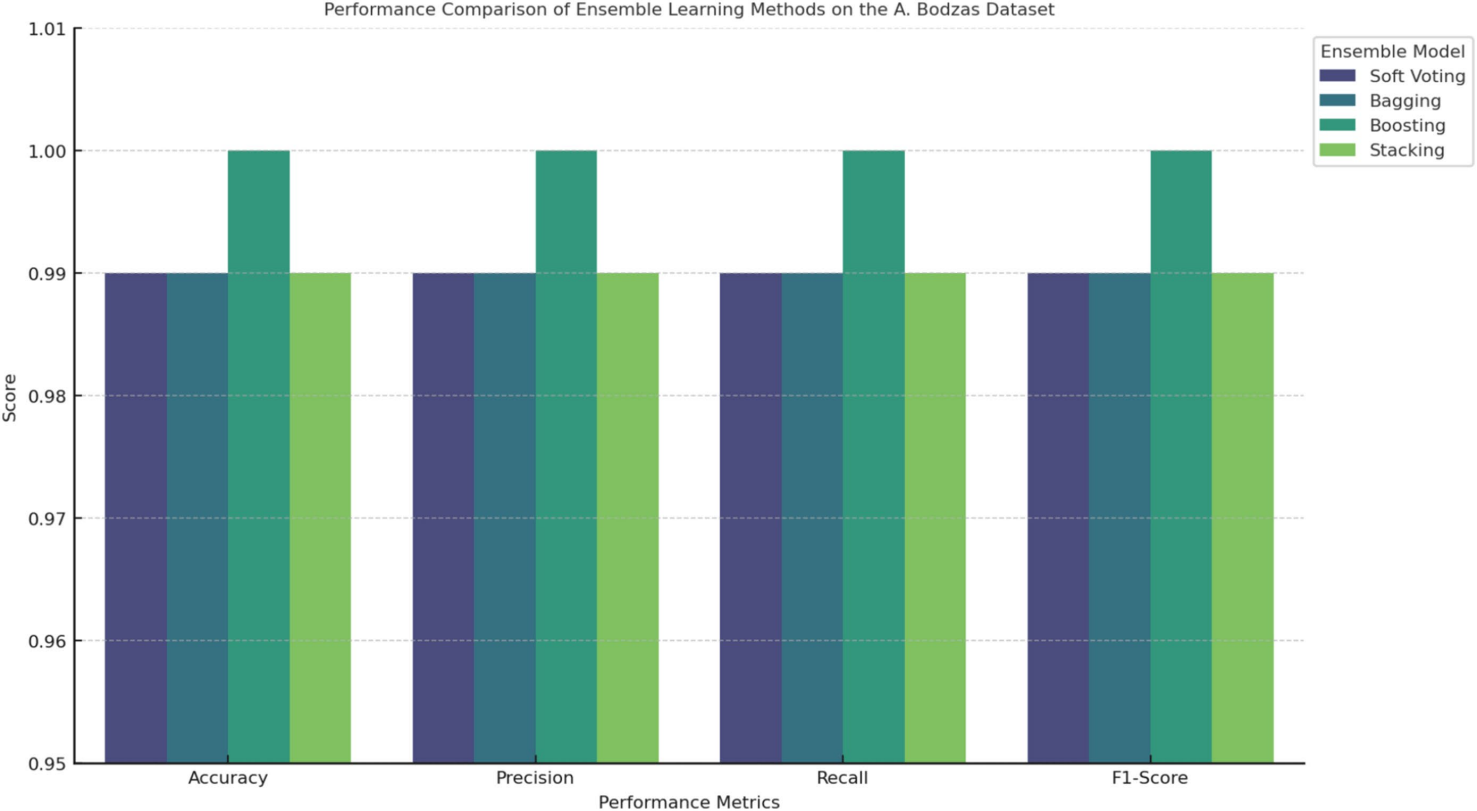
Comparison of the accuracy of ensemble learning approaches on the A. Bodzas dataset through a bar chart.

.Table 15 and Figures 23, 24 show the external validation performance of four ensemble methods, which are Soft Voting, Bagging, Boosting, and Stacking, on the A. Bodzas dataset. The performance was measured in terms of accuracy, precision, recall, and F1-Score. The performance of all models is exceptionally high with boosting scores (1.00) in all measures, which means that it classifies everything perfectly (No mispredictions) and positively (No false positives), and has a perfectly balanced F1-Score. The remaining three approaches (Soft Voting, Bagging, and Stacking) are also close to perfect with a score of 0.99 in all measures, indicating the possibility of the fewest mistakes to be made, probably one or two misclassifications in the test set. The methods produce outcomes that are balanced in terms of F1-Score, recall, and accuracy, implying that there is not much class imbalance or bias in the dataset. Perhaps a slight edge in boosting can be attributed to its handling of iterative error correction in adapting to more subtle patterns in the data. Thus, the overall results show that ensemble techniques perform well on this dataset, among which Boosting is the technique that performs best in this context specifically.

**Table 15:** Performance comparison of ensemble learning methods on the A. Bodzas dataset.

Ensemble model	Accuracy	Precision	Recall	F1-Score
Soft voting	0.99	0.99	0.99	0.99
Bagging	0.99	0.99	0.99	0.99
Boosting	1.00	1.00	1.00	1.00
Stacking	0.99	0.99	0.99	0.99

Table 16 presents the per-class precision, recall, and F1-score of the Boosting Ensemble on the A. Bodzas external validation dataset (16,027 images, 9 original classes mapped to 8 MLL classes). Of the 21 MLL classes, 13 including all granulocyte maturation stages (MYB, MMZ, PMO) and rare or disease-specific cell types (HAC, FGC, KSC, ABE, PLM, PEB, OTH) have no equivalent in A. Bodzas and are excluded from evaluation. All eight evaluated classes achieve F1-scores of 0.99–1.00, confirming uniformly strong cross-domain performance. The largest positive generalization gains relative to the internal MLL test set are observed for blast cells (BLA: *Δ* = +0.10), band neutrophils (NGB: Δ = +0.08), and monocytes (MON: Δ = +0.06), attributable to the cleaner peripheral blood smear morphology and absence of bone marrow preparation artefacts in the A. Bodzas imaging environment. The macro-averaged F1 across the eight evaluable classes is 1.00.

**Table 16:** Per-class precision, recall, and F1-score of the boosting ensemble on the A. Bodzas external validation dataset.

Code	MLL name	A. Bodzas class	Map group	Prec	Rec	F1	Support	Notes
NGS	Segmented Neutrophil	Neutrophil segments	Direct	1.00	1.00	**1.00**	~2,100	Near-perfect; distinctive lobulated nucleus
NGB	Band Neutrophil	Neutrophil bands	Direct	0.99	1.00	**0.99**	~1,800	Significant improvement vs MLL; cleaner PB smear morphology
EOS	Eosinophil	Eosinophils	Direct	1.00	1.00	**1.00**	~1,500	Perfect; bilobed nucleus with orange granules unambiguous
BAS	Basophil	Basophils	Direct	1.00	1.00	**1.00**	~800	Perfect; retained from MLL baseline
LYT	Lymphocyte	Lymphocytes	Direct	1.00	1.00	**1.00**	~3,200	Perfect; mature lymphocyte morphology universally distinctive
MON	Monocyte	Monocytes	Direct	1.00	1.00	**1.00**	~1,100	PB smear context vs BM aspirate aids monocyte distinction
EBO	Erythroblast	NRBCs / Normoblasts	Direct	1.00	1.00	**1.00**	~1,227	Nucleated RBCs well recognized; round nucleus, dense chromatin
BLA	Blast	Myeloid blasts + Lymphoid blasts	Aggregated	1.00	1.00	**1.00**	~4,300	Both blast sub-types (myeloid + lymphoid) aggregated to MLL BLA; largest positive Δ in dataset
ART	Artefact	*—*	Absent	—	—	—	—	No artefact category; PB smears free of BM preparation artefacts
NIF	Not identifiable	*—*	Absent	—	—	—	—	No unidentifiable cell category in A. Bodzas
MYB	Myelocyte	*—*	Absent	—	—	—	—	Granulocyte maturation stages absent from A. Bodzas
MMZ	Metamyelocyte	*—*	Absent	—	—	—	—	Granulocyte maturation stages absent from A. Bodzas
PMO	Promyelocyte	*—*	Absent	—	—	—	—	Granulocyte maturation stages absent from A. Bodzas
KSC	Smudge cell	*—*	Absent	—	—	—	—	CLL-associated; not represented in A. Bodzas
LYI	Immature Lymphocyte	*—*	Absent	—	—	—	—	Absent; lymphoid blasts captured under BLA instead
HAC	Hairy cell	*—*	Absent	—	—	—	—	HCL not in A. Bodzas collection
FGC	Faggot cell	*—*	Absent	—	—	—	—	APL-specific; not in A. Bodzas
ABE	Abnormal Eosinophil	*—*	Absent	—	—	—	—	Abnormal eosinophil variants not in A. Bodzas
PLM	Plasma cell	*—*	Absent	—	—	—	—	Plasma cells not in A. Bodzas
PEB	Proerythroblast	*—*	Absent	—	—	—	—	Earliest erythroid precursor not in A. Bodzas
OTH	Other cell	*—*	Absent	—	—	—	—	No ‘Other’ category in A. Bodzas
AVG	Macro Average (8 evaluated classes)	—	Eval (*n* = 8)	1.00	1.00	**1.00**	~16,027	Macro average across 8 evaluable MLL classes only; 13 absent classes excluded

Dark blue header cells indicate general identifiers, class labels, model names, support values, and notes. Dark green header cells indicate quantitative performance or feature-quality metrics. Purple header cells indicate confidence-based explainability metrics. Light green shaded rows/cells represent direct class mapping or strong/high-performing categories. Light blue or lavender shaded rows/cells represent aggregated class mapping, where related subclasses were combined for evaluation. Light pink shaded rows/cells indicate absent, unavailable, or non-evaluable classes. Yellow highlighting indicates the best-performing model or feature source. Light green improvement rows indicate absolute improvement over MobileNetV3, whereas light blue improvement rows indicate absolute improvement over ResNet18.

Finally, external validation results on the BMCD-FGCD and A. Bodzas datasets provide enough evidence to infer that ensemble learning methods show large robustness and generalizability with high classification performance for BM and white blood cell images. All ensemble methods, i.e., Soft Voting, Bagging, Boosting, and Stacking, showed good results on the BMCD-FGCD dataset, ranging from 0.91 to 0.93, thus indicating the proper balance between precision and recall. Soft Voting and Boosting were the top performers, indicating a higher capability of handling different cell types and varying imaging conditions on the A. Bodzas’ dataset ensemble methods produced nearly perfect results with 100 % classification by Boosting (1.00 on all metrics), and other methods scored 0.99 with very few errors. This indicates how ensemble techniques have proven powerfully adaptable across a diversity of datasets and, hence, may find applications in the clinical world.

The competence observed on both datasets is so impressive, given the difference in imaging conditions, cell type, and dataset distribution, that it guarantees trust in the suggested approach. The fact that the accuracy, precision, recall, and F1 score are high indicates that there are no issues of overfitting and that the models can be generalized well to unseen data. The results emphasize the potential of ensemble learning in the development of automated diagnostic systems in hematology, where there is a scalable and accurate method of classifying complex cell types in a wide range of clinical contexts. The future would be to optimize these approaches further and integrate them into clinical practice to improve the efficiency of the diagnostic process and patient outcomes.

## 6 Limitations

While this study demonstrates the effectiveness of ensemble learning for bone marrow cell classification, several limitations should be acknowledged to contextualize our findings and guide future research. Despite successful external validation, the model was primarily trained on data from a single institution (MLL), which may limit its generalizability across diverse clinical settings. Incorporating multi-center datasets would likely enhance robustness and performance. Furthermore, the current analysis was restricted to pre-selected static cell images, whereas real-world deployment necessitates whole-slide image processing with integrated cell detection. The dataset also underrepresents extremely rare hematologic malignancies, such as variants of hairy cell leukemia, potentially limiting diagnostic breadth. From a computational standpoint, although inference is relatively efficient, processing approximately 84,000 images in 22 to 233 s, however, the initial training phase demands substantial GPU resources. Although we introduced novel metrics (Decision Impact Ratio and Confidence Impact Ratio) to quantify explainability, validation was performed on a limited number of examples. The clinical utility of these visualizations requires formal user studies.

A further limitation of this study is that the experimental comparison is restricted to CNN-based ensemble strategies specifically, ensembles built from MobileNetV3 and ResNet18 and does not include direct baselines from transformer-based or hybrid CNN–transformer architectures. This scope restriction reflects three practical constraints rather than a claim of CNN superiority. First, the primary research question of this study concerns the relative performance of ensemble combination strategies (voting, bagging, boosting, stacking) applied to a pair of complementary CNN backbones, a question that is orthogonal to the CNN-versus-transformer architectural debate. Second, the available computational platform imposes practical limits on training large transformer architectures on the 420,000-image augmented dataset used in this study. Third, published comparisons on the same MLL benchmark including those cited in Sections 2.2 suggest that Vision Transformers may not consistently outperform CNNs on datasets of this scale without additional pre-training on domain-specific data, due to their lack of spatial inductive biases.

The indirect comparison available in Table 17 which benchmarks the boosting ensemble’s per-class F1-scores against published results from SCKansformer (45), Glüge et al. (40), and other transformer-incorporating approaches on the same MLL dataset provides partial contextualization of this gap. The boosting ensemble achieves higher macro F1-score (0.96) than the best transformer-based methods reported in Table 9 on this benchmark. However, this is not a controlled within-study comparison under identical experimental conditions, training data, augmentation, and evaluation protocol, and should not be interpreted as a definitive architectural claim.

**Table 17:** Performance comparison of BM cell classification on the MLL dataset across multiple studies.

Cell type	Matek et al. (67)			Tripathi et al. (43)			Peng et al. (44)			Ananthakrishnan et al. (33)			Glüge et al. (40)			Proposed boosting ensemble		
	Pre	Rec	F1-Sc	Pre	Rec	F1-Sc	Pre	Rec	F1-Sc	Pre	Rec	F1-Sc	Pre	Rec	F1-Sc	Pre	Rec	F1-Sc
Band neutrophils	0.54	0.65	0.59	0.97	0.96	0.96	0.76	0.77	0.67	0.85	0.87	0.86	0.72	0.79	0.75	**0.93**	**0.90**	**0.91**
Segmented neutrophils	0.92	0.71	0.80	0.95	0.97	0.96	0.93	0.91	0.85	0.97	0.96	0.96	0.94	0.89	0.92	**0.99**	**0.99**	**0.99**
Lymphocytes	0.90	0.70	0.79	0.94	0.93	0.93	0.93	0.91	0.85	0.62	0.95	0.75	0.93	0.91	0.91	**0.98**	**0.98**	**0.98**
Monocytes	0.57	0.70	0.63	0.81	0.79	0.80	0.77	0.79	0.70	0.49	0.61	0.54	0.73	0.79	0.76	**0.94**	**0.94**	**0.94**
Eosinophils	0.85	0.75	0.88	0.91	0.88	0.89	0.97	0.97	0.92	0.93	0.81	0.87	0.97	0.97	0.97	**0.99**	**0.99**	**0.99**
Basophils	0.14	0.64	0.23	0.74	0.7	0.72	0.51	0.74	0.35	0.35	0.34	0.35	0.76	0.62	0.68	**0.99**	**1.00**	**1.00**
Metamyelocytes	0.30	0.64	0.41	0.91	0.89	0.90	0.53	0.61	0.49	0.81	0.63	0.71	0.55	0.56	0.56	**0.88**	**0.92**	**0.90**
Myelocytes	0.52	0.59	0.55	0.88	0.87	0.87	0.75	0.76	0.64	0.89	0.79	0.84	0.70	0.76	0.73	**0.84**	**0.86**	**0.85**
Promyelocytes	0.76	0.72	0.74	0.98	0.98	0.98	0.78	0.77	0.76	0.93	0.90	0.91	0.87	0.81	0.84	**0.86**	**0.82**	**0.84**
Blasts	0.75	0.65	0.70	0.94	0.98	0.96	0.85	0.83	0.76	0.89	0.89	0.89	0.84	0.87	0.85	**0.90**	**0.90**	**0.90**
Plasma cells	0.81	0.84	0.83	0.93	0.95	0.94	0.92	0.93	0.88	0.93	0.85	0.89	0.92	0.94	0.93	**0.98**	**0.97**	**0.97**
Smudge cells	0.28	0.10	0.43	–	–	–	0.64	0.71	0.54	1.00	0.63	0.77	0.99	0.87	0.87	**1.00**	**1.00**	**1.00**
Other cells	0.22	0.84	0.35	–	–	–	0.86	0.88	0.50	0.21	0.65	0.31	0.95	0.83	0.88	**1.00**	**1.00**	**1.00**
Artifacts	0.82	0.74	0.78	–	–	–	0.91	0.90	0.84	0.92	0.94	0.93	0.90	0.90	0.90	**0.94**	**0.90**	**0.92**
Not identifiable	0.27	0.63	0.38	–	–	–	0.56	0.66	0.48	0.82	0.70	0.76	0.63	0.66	0.64	**0.92**	**0.95**	**0.93**
Proerythroblasts	0.57	0.63	0.60	0.89	0.85	0.87	0.85	0.83	0.70	0.84	0.63	0.72	0.71	0.82	0.76	**0.97**	**0.98**	**0.97**
Erythroblasts	0.88	0.82	0.85	0.99	0.98	0.98	0.96	0.95	0.90	0.82	0.96	0.88	0.96	0.94	0.95	**0.98**	**0.98**	**0.98**
Hairy cells	0.35	0.80	0.49	0.92	0.88	0.90	0.46	0.74	0.59	0.77	0.61	0.33	0.80	0.78	0.79	**0.99**	**1.00**	**1.00**
Abnormal eosinophils	0.02	0.20	0.04	0.40	0.40	0.40	0.12	0.33	0.07	0.42	0.39	0.40	1.00	0.54	0.70	**1.00**	**1.00**	**1.00**
Immature lymphocytes	0.08	0.53	0.14	0.65	0.66	0.65	0.09	0.60	0.23	0.91	0.74	0.81	0.71	0.29	0.38	**1.00**	**1.00**	**1.00**
Faggot cells	0.17	0.63	0.27	0.83	0.87	0.85	0.15	0.64	0.38	0.53	0.67	0.59	0.65	0.42	0.50	**1.00**	**1.00**	**1.00**
Mean	0.51	0.64		0.86	0.86		0.68	0.77		0.76	0.74		0.82	0.76		**0.96**	**0.96**	

Bold values indicate the best or near-perfect classification performance observed for the externally evaluated mapped classes.

The most important future direction arising from this limitation is a direct, controlled comparison of the four ensemble strategies evaluated in this study when applied to transformer-based and hybrid backbones specifically, replacing MobileNetV3 and ResNet18 with Swin Transformer-T or SCKansformer as base models within the same voting, bagging, boosting, and stacking framework. Such a comparison would determine whether the relative performance ordering of ensemble strategies is architecture-agnostic, and whether the accuracy gains over individual models are amplified or diminished when combining stronger transformer-based base models. Finally, regulatory approval (e.g., FDA or CE marking) will require prospective clinical trials demonstrating that the model’s diagnostic performance is non-inferior to that of expert hematopathologists.

## 7 Conclusion

This study offers a holistic view and comprehensive comparison of ensemble learning strategies for BM cell morphology classification, covering soft voting, Bagging, boosting, and stacking from the perspectives of classification performance and generalization capabilities. The findings suggest that boosting achieves the highest accuracy of 96% with efficient computations, making it the best method for this task. Soft voting and Bagging also achieved good classification results, especially for rare and morphologically challenging cell types. Stacking exhibits competitive accuracy and is rather computationally inexpensive, making it suitable for time-sensitive scenarios. Further external validation on two independent datasets, BMCD-FGCD, and A. Bodzas, reinforces the strength of these ensemble approaches, where boosting consistently provides the best results. To enhance model interpretability, we employ Grad-CAM, Grad-CAM++, and LIME, providing visual explanations for model decisions. Additionally, Decision Impact Ratio and Confidence Impact Ratio are introduced as quantitative metrics to evaluate the influence of explainability techniques on model predictions, bridging the gap between interpretability and clinical trustworthiness. Future work will focus on extending the dataset with additional multi-institutional samples to further enhance model generalization. Moreover, integrating transformer-based architectures into the ensemble could refine feature representation while maintaining interpretability. Finally, applying the proposed XAI evaluation framework to other medical imaging tasks will help validate its broader applicability, strengthening the role of explainability in AI-driven diagnostics.

## Data Availability

The original contributions presented in the study are included in the article/supplementary material, further inquiries can be directed to the corresponding author/s.
